# Field validation of deep learning based Point-of-Care device for early detection of oral malignant and potentially malignant disorders

**DOI:** 10.1038/s41598-022-18249-x

**Published:** 2022-08-22

**Authors:** Praveen Birur N., Bofan Song, Sumsum P. Sunny, Keerthi G., Pramila Mendonca, Nirza Mukhia, Shaobai Li, Sanjana Patrick, Shubha G., Subhashini A.R., Tsusennaro Imchen, Shirley T. Leivon, Trupti Kolur, Vivek Shetty, Vidya Bhushan R., Daksha Vaibhavi, Surya Rajeev, Sneha Pednekar, Ankita Dutta Banik, Rohan Michael Ramesh, Vijay Pillai, Kathryn O.S., Petra Wilder Smith, Alben Sigamani, Amritha Suresh, Rongguang Liang, Moni A. Kuriakose

**Affiliations:** 1 Karnataka Lingayat Education Society’s Institute of Dental Sciences, Bangalore, India; 2 Integrated Head and Neck Oncology Program, Mazumdar Shaw Medical Foundation, Narayana Health City, Bommsandra Industrial Area, Bangalore, 99 India; 3 Biocon Foundation, Bangalore, India; 4 College of Optical Sciences, The University of Arizona, Tucson, AZ USA; 5 Department of Head and Neck Surgical Oncology, Mazumdar Shaw Medical Center, Narayana Health City, Bangalore, India; 6 Christian Institute of Health Sciences and Research, Dimapur, Nagaland India; 7 Beckman Laser Institute, University of California Irvine School of Medicine, Irvine, USA; 8 Clinical Research, Mazumdar Shaw Medical Center, Bangalore, India; 9 Present Address: Karkinos Healthcare, Kochi, India

**Keywords:** Cancer imaging, Cancer prevention, Cancer screening, Head and neck cancer, Oral cancer, Cancer, Oral diseases, Image processing, Machine learning, Clinical trial design, Translational research

## Abstract

Early detection of oral cancer in low-resource settings necessitates a Point-of-Care screening tool that empowers Frontline-Health-Workers (FHW). This study was conducted to validate the accuracy of Convolutional-Neural-Network (CNN) enabled m(mobile)-Health device deployed with FHWs for delineation of suspicious oral lesions (malignant/potentially-malignant disorders). The effectiveness of the device was tested in tertiary-care hospitals and low-resource settings in India. The subjects were screened independently, either by FHWs alone or along with specialists. All the subjects were also remotely evaluated by oral cancer specialist/s. The program screened 5025 subjects (Images: 32,128) with 95% (n = 4728) having telediagnosis. Among the 16% (n = 752) assessed by onsite specialists, 20% (n = 102) underwent biopsy. Simple and complex CNN were integrated into the mobile phone and cloud respectively. The onsite specialist diagnosis showed a high sensitivity (94%), when compared to histology, while telediagnosis showed high accuracy in comparison with onsite specialists (sensitivity: 95%; specificity: 84%). FHWs, however, when compared with telediagnosis, identified suspicious lesions with less sensitivity (60%). Phone integrated, CNN (MobileNet) accurately delineated lesions (n = 1416; sensitivity: 82%) and Cloud-based CNN (VGG19) had higher accuracy (sensitivity: 87%) with tele-diagnosis as reference standard. The results of the study suggest that an automated mHealth-enabled, dual-image system is a useful triaging tool and empowers FHWs for oral cancer screening in low-resource settings.

## Introduction

Oral cancer is a rising global health problem, with the highest incidence being reported in the Indian sub-continent^1,2^. Stage at diagnosis is the primary determinant of its oncologic outcome^1^. Eighty percentage of oral cavity cancers in a high-prevalent population are preceded by asymptomatic, clinically evident lesions, referred to as oral potentially malignant disorders (OPMD)^3^. Early detection and management at this stage can significantly decrease the mortality rate^4^. However, despite well-defined clinical diagnostic features and easy accessibility, over 70% of oral cancers are diagnosed at advanced stages^5^. This delay is attributed to the late presentation by the patients and the inability of the primary health care providers to identify suspicious OPMD^6^. The current diagnostic pathway, which includes visual examination-based detection of suspicious lesions by the primary health care providers, followed by incisional biopsy^7,8^, is not feasible for the large-scale screening of high-risk populations^9^.

Diagnostic adjuncts are known to improve the efficacy of oral lesion detection in the primary health care settings. Vital dyes, auto-fluorescence, chemiluminescence, Raman spectroscopy, optical-coherence-tomography, and biomarker-based imaging have been explored for early detection of oral cancer^10–12^. However, they are often limited by poor efficacy in discrimination of benign lesions from OPMD^10,12^. In addition, affordability and the need for specialist interpretation lower its adoption^11,13^. An effective oral cancer screening necessitates robust, simple, automated, Point-of-Care (PoC) tools that empower Frontline-Health-Workers (FHW) to effectively triage large-volume, high-risk populations^14–16^.

Previous studies^17^, including those from our group, have demonstrated the immense potential of digital, PoC imaging systems, enabling access to remote specialists, in the detection of oral cancer^15,18^. Auto-fluorescence Imaging (AFI) in combination with automated diagnosis and specialist interpretation of the images, have shown promising results in oral cancer screening^19–21^.Our team has previously reported the feasibility of a dual-mode imaging device, combining AFI and White Light Imaging (WLI) for oral cancer diagnosis^16^. The primary aim of this study was to evaluate the clinical efficacy of the artificial intelligence (AI) enabled, dual-mode imaging device for oral cancer screening as in low-resource-settings. The device was assessed for its efficacy as a PoC system to improve the accuracy of FHW-based screening, as a telediagnosis tool and to detect OPMD and oral cancer in the community.

## Results

### Demographics and clinical information

**Figure 1:**
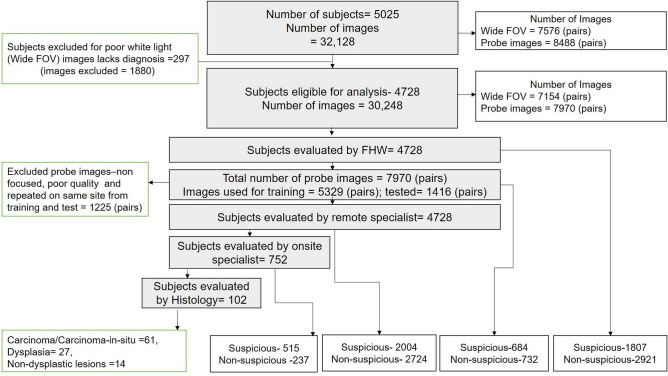
Study consort chart. A total of 5025 subjects were recruited for study according to inclusion and exclusion criteria. From 5025 subjects, a total of 32,128 images were captured using the phone with wide FOV (WLI and AFI = 7576 pairs) and probe with the focused view (WLI and AFI = 8488 pairs). More images were recorded of the same lesions using the probe. Among the subjects, 297 cases were not interpretable (WLI) by remote specialists and hence excluded from further analysis. All the subjects (n = 4728) were directly visualized by FHW and assessed by the remote specialist. The probe images were used for training/cross-validation (n = 5329 pairs) and testing (n = 1416 pairs). Out of 4728, 16% (n = 752) cases were seen directly by the onsite specialist. Onsite specialists identified 515 cases as suspicious and 20% (n = 102) underwent incisional biopsy and histology evaluation. Remote specialists and FHWs identified a suspicious lesion in 2004 and 1807 subjects respectively. *FOV* field of view, *FHW* Front-Line-Health Worker, *WLI* White Light Imaging, *AFI* Auto-fluorescence Imaging.

A total of 5025 subjects (images: n = 32,128) were accrued in the study (September 2019 to March 2020). Among the subjects, 297 (6%) cases were not interpretable by remote specialists and hence excluded from the study (Fig. 1). The gender (M: 52%; F: 48%) and age distribution (median age: 53 years) were matched across the different study centers. Tobacco and/or areca nut chewers constituted 62% (n = 2919) of the subjects, while 18% (n = 860) were tobacco smokers. Among the subjects accrued from the Christian Institute of Health Sciences and Research (CIHSR), majority were either unemployed (37%) or from the student community (20%), while in the clusters from Karnataka Lingayat Education Institute of Dental Sciences (KLE) majority were self-employed or home-makers (68%) (Supplementary Fig. S1a–h).

All the subjects included in the study (n = 4728) were directly evaluated by FHWs and telediagnosis by remote specialists. Sixteen percent (n = 752) of the subjects were assessed by onsite specialists. FHWs predominantly detected white (n = 1286/4728) or white-red patches (326/4728). Remote specialists and FHWs diagnosed 42% (2004/4728) and 38% (1807/4728) respectively as suspicious lesions. Out of the 2004 subjects deemed suspicious and referred for further evaluation by remote specialists, 752 subjects were evaluated by the onsite specialists. The onsite specialist identified 69% (515/752) as suspicious lesions or indicated for biopsy and referred to tertiary care facilities. Twenty percent (102/515) underwent the biopsy procedure (Fig. 1).

### Comparison of onsite specialists’ diagnosis to histology

The efficacy of the onsite specialists was assessed in comparison with histology (n = 102). The lesions detected in this cohort were diagnosed as oral squamous cell carcinoma or carcinoma-in-situ (n = 61), dysplasia (n = 27), and non-dysplasia (n = 14). The onsite specialists showed a high sensitivity of 94% (CI 86.76–97.65) and specificity of 72% (CI 29.04–96.33) for diagnosis of OPMD and oral cancer with histology as the gold standard (Table 1).

**Table 1:** Comparison of the different tests according to the reference standard.

	Onsite visual examination by specialist vs histology diagnosis	Remote diagnosis vs onsite visual examination by specialist	Onsite visual examination by FHW vs remote diagnosis by specialist	Onsite visual examination by FHW vs onsite diagnosis by specialist	Diagnosis by by MobileNET (in mobile phone) vs remote diagnosis by specialist	Diagnosis VGG19 (in cloud, BDL uncertainty < 0.15) vs remote diagnosis (specialist)
True positive	89	489	1203	507	493	420
True negative	5	198	2120	124	622	591
False positive	2	39	604	113	191	94
False negative	6	26	801	8	110	62
Total	102	752	4728	752	1416	1167
Sensitivity	94%	95%	60%	98%	82%	87%
Specificity	72%	84%	78%	52%	77%	86%
PPV	98%	93%	67%	82%	72%	82%
NPV	46%	88%	73%	94%	85%	90%
Accuracy	93%	92%	70%	84%	79%	87%
Prevalence of OPMD/oral cancer (reference standard)	93% (95/102)	69% (515/752)	42% (2004/4728)	69% (515/752)	43% (603/1416)	41% (482/1167)
Weighted Kappa	0.52 (0.22–0.81)	0.79 (0.75–0.84)	0.38 (0.36–0.41)	0.58 (0.5151–0.64)	0.57 (0.53–0.62)	0.76 (0.72–0.80)

The table depicts the number of true positives, true negatives, false positives, false negatives, and total cases. The sensitivity, specificity, positive predictive value (PPV), and negative predictive value (NPV), and accuracy were calculated according to the reference standard. The kappa statistics, to find the agreement, was performed between the reference standard and test. *OPMD* Oral Potentially Malignant Disorders, *FHW* Front line health worker.

### Efficacy of dual-modality imaging device as a telediagnosis tool

The tele-diagnostic efficacy of the device was assessed in two ways (i) image quality assessment for remote interpretation and (ii) accuracy of the remote specialist. Natural Image Quality Evaluator (NIQE) based assessment of the WLI on the wide field of view (FOV) used for remote diagnosis (Supplementary Fig. S2), demonstrated that both the phone (average score: 4.45; CI 4.44–4.46) and probe (Score: 8.09; CI 8.17–8.23) images were of good quality. The NIQE image quality score (Supplementary Fig. S2c) showed significant (p < 0.001) improvement in the second quadrant (7.97 ± 0.02) of the study compared to the first (8.51 ± 0.03) indicating the benefits of regular training of the FHWs.

The remote specialists showed a sensitivity and specificity of 95% (CI 92.69–96.68) and 84% (CI 78.2–88.03) in diagnosis of OPMD and oral cancer respectively, when compared to the onsite specialist (n = 752) with an agreement of 0.79 (CI 0.75–0.844) (Table 1).

### Diagnostic efficacy of FHW in comparison with onsite and remote specialist

The accuracy of FHWs was assessed in terms of the benefits of training (comparison to onsite specialist) and tele-diagnosis (comparison with remote specialist). Analysis of the accuracy of FHW in the two clusters (with and without onsite specialist) separately indicated that FHWs in the team with an onsite specialist showed a high sensitivity of 98% (CI 92.08–95.84) and low specificity of 52.41% (CI 46.70–58.08). However, the FHWs in the team without an onsite specialist showed a comparatively low sensitivity (43.61%; CI 41–46.22) and high specificity of 81% (CI 79.45–82.61), emphasizing the need for regular training of FHWs to improve sensitivity. Multi-variate analysis also showed that the FHWs with onsite specialists (NH1, NH2, and KLE1, 2) showed a better performance (highest F1 score) (Supplementary Fig. S3a) as compared to those in the team without specialists.

Direct visualization by FHWs demonstrated a moderate sensitivity of 60% (CI 57.85–62.18) and a specificity of 78% (76.22–79.37) in delineating suspicious lesions (Table 1), when compared with the telediagnosis. Further, multivariate analysis (Supplementary Fig. S3, Table S1) to identify parameters contributing towards FHW diagnosis (F1 score calculated due to an imbalance in subject recruitment), indicated a dependence on experience in the current project. This factor showed a high positive correlation with the F1 Score (r = 0.80; 0.54–0.92; p = 0.028) (Supplementary Fig. S3b, Table S1). These results indicated the need for skilled expertise and adjuncts for improving accuracy of FHW diagnosis.

### Effectiveness of CNN-integrated dual mode imaging device as a Point-of-Care (POC) diagnostic tool

**Figure 2:**
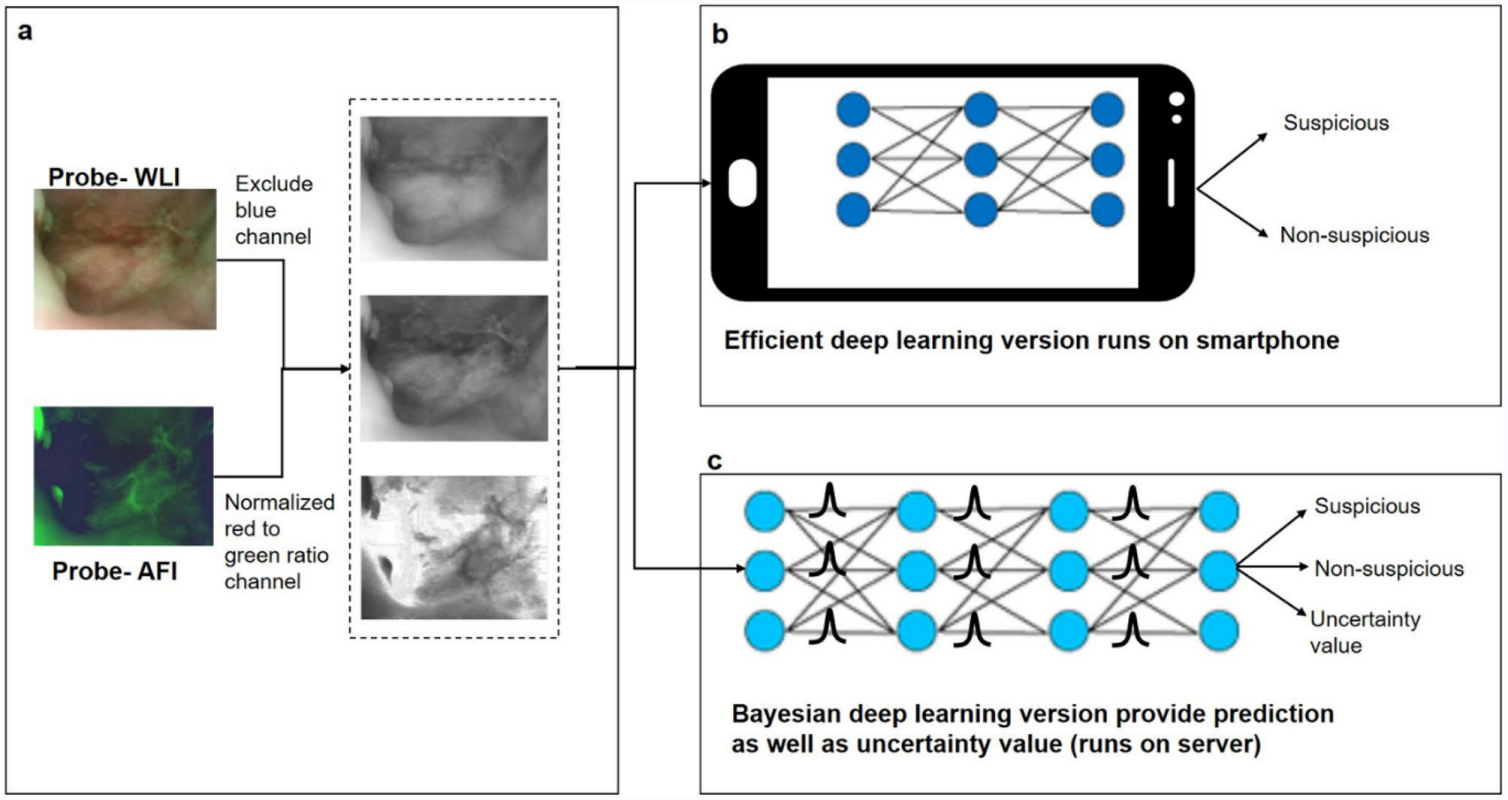
Pipeline of artificial intelligence-based image classification. The feature extraction of probe WLI and AFI was performed using green and red channels of WLI and the normalized ratio of AFI-red/green channels (**a**). These images were fused to feed the neural networks for real-time analysis in phones and also for cloud systems. The efficient version of Convolutional Neural Network (CNN) was built which runs on a smartphone device (**b**) classified images as suspicious or non-suspicious. Another more complex CNN based on Bayesian deep learning framework was built (**c**) for predicting uncertainty along with diagnosis in a cloud system. *CNN* Convolution Neural Network, *WLI* White Light Imaging, *AFI* Auto-fluorescence Imaging)**.**

The image dataset (probe images = 5329) was randomly split into training (75%) and cross-validation (25%) with an additional standalone test dataset (n = 1416). We trained two different versions of deep learning models (Fig. 2, Supplementary Fig. S4); one deployed on the smartphone, and the BDL model deployed on the cloud server. The MobileNet integrated with the mobile phone detected suspicious lesions in real-time with 82% (CI 78.44–84.76) sensitivity and 77% (CI 73.44–79.38) specificity, when compared to remote diagnosis. Further classification of the lesions indicated that MobileNet could delineate OSCC (Sensitivity: 94%; CI 73–98.97) and OPMD (Sensitivity: 81%; CI 73.54–81.87) from benign lesions and normal mucosa. A comparison of the performance of MobileNet with InceptionV3 and VGG19 CNN models (Supplementary Table S2), indicated a significant reduction in the number of parameters and the model size with minimal compromise in accuracy.

The BDL, with a more complex neural network, achieved 85% (CI 81.44–87.37) sensitivity and 82% (CI 79.62–84.97) specificity on the standalone dataset. An uncertainty threshold of 0.15 improved the accuracy (85% to 87%) with 18% of the patients needing reference, this was hence chosen as the cut-off (Table 1, Supplementary Figs. S4, S5). In case of uncertainty (> 0.15), a remote diagnosis of the images could be carried out. The final model including the BDL model in comparison with remote diagnosis achieved a sensitivity of 87% (CI 83.82–89.99) and specificity of 86% (83.47–88.77).

## Discussion

**Figure 3:**
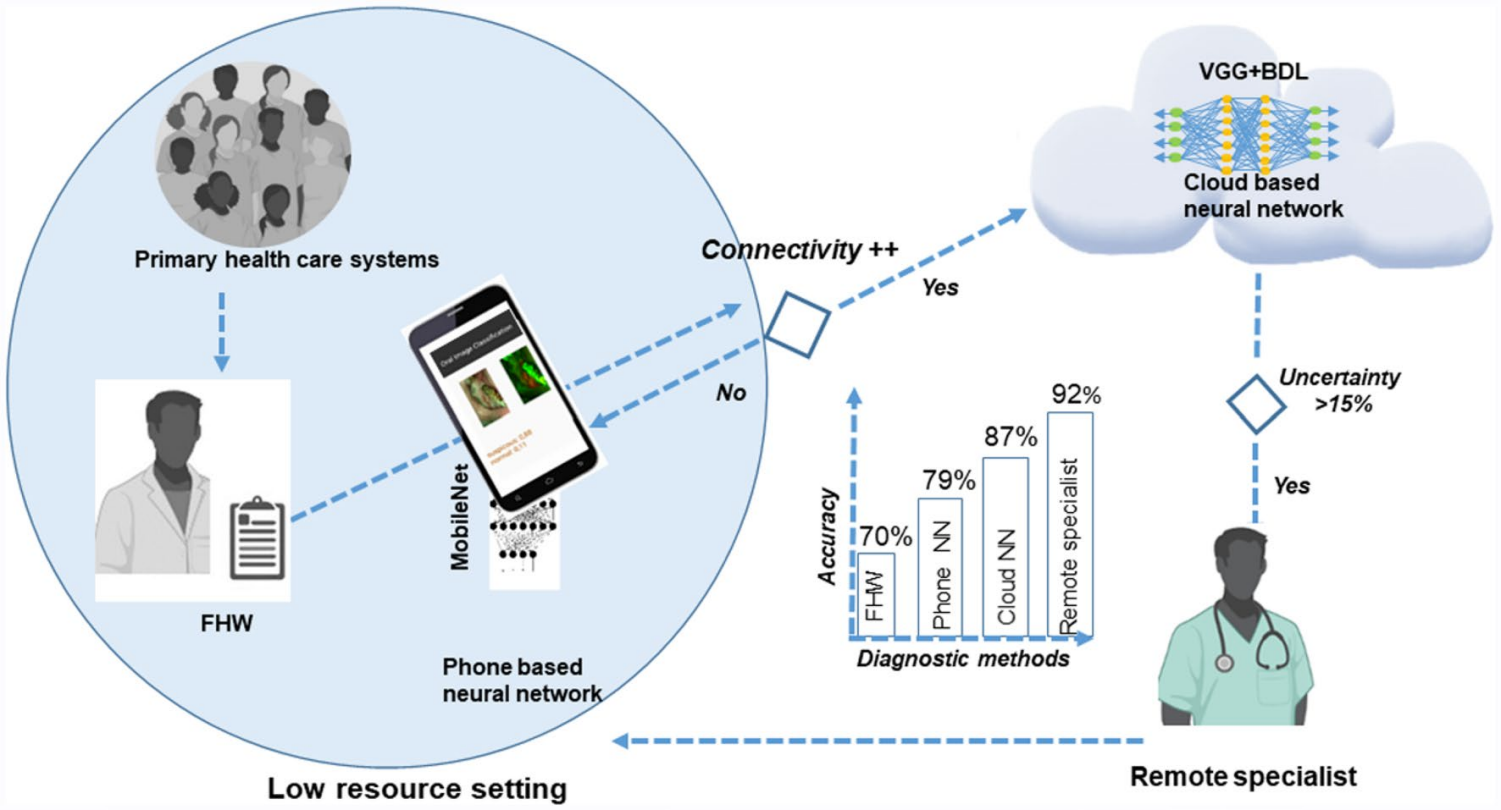
Diagnostic model in Low resource setting. The study depicted that a dual-mode imaging system deployed with Front Line Health workers (FHW) improved the diagnostic efficacy in delineating suspicious oral potentially malignant and malignant lesions. In low resource settings with poor internet connectivity, the mobile phone-based neural network, MobileNet, which had an accuracy of 79%, can be used. If connectivity is good (++), a more accurate cloud-based neural network (VGG19-BDL, accuracy = 87%), should be used and difficult cases with high uncertainty value should be referred to remote specialists (accuracy = 92%) for interpretation. *NN* Neural Network, *FHW* Front-Line-Health-Workers, *VGG19-BDL*: VGG19 Convolution Neural Network with Bayesian prediction. Images Primary health care systems, *FHW*,Remote specialist were created with “BioRender.com” (https://biorender.com) and Cloud based neural network with Microsoft Paint Windows.

Population-based screening of oral cancer through visual examination by trained FHWs is established as a cost-effective approach, reducing the mortality rate by 34%^7^. Studies have demonstrated the feasibility of trained FHWs in identifying oral lesions^7,14,18,22^, however, scalability was a concern, due to the inability to consistently train manpower. This study, to the best of our knowledge, is a first of its kind that deployed a PoC diagnostic tool to empower FHWs in a community-based screening program, for early detection of oral cancer. The findings of the study suggest that this portable imaging device empowered the FHWs for accurate screening and surveillance of oral cancer in a resource-constrained setting (Table 1). The sensitivity of the device in tele-diagnosis (95%) and in PoC diagnosis (82%), were comparable to the direct examination by onsite specialists, indicating the feasibility of the approach as a potential oral cancer screening strategy in resource-constrained, high-risk populations (Fig. 3).

Visual examination-based detection of suspicious lesions by onsite specialist followed by incisional biopsy forms the current oral cancer/OPMD diagnostic pathway. Tele-diagnosis is a valuable approach to provide specialist expertise to low resource settings. A previous study from our group compared remote specialist diagnosis (using a mobile phone) with onsite diagnosis; remote diagnosis achieved the sensitivity and specificity of 96.2% and 97.6% respectively in delineation of suspicious lesion when compared to onsite diagnosis^14^. In the current study, visual examination by onsite specialists was comparable to histology diagnosis in the detection of OPMD/oral cancer. The dual-mode device, evaluated in the study, was also effective in telediagnosis, with remote diagnosis being comparable to the onsite diagnosis for identification of suspicious lesions [Table 1; Kappa 0.79 CI (0.75–0.84)]. However, direct FHW diagnosis achieved only a moderate sensitivity (60%), indicating that while onsite, remote diagnosis and histology are comparable (Table 1; Kappa 0.52–0.78), an independent FHW-based, large-scale screening in low-resource settings, necessitates diagnostic adjuncts for improving accuracy.

The current PoC devices although aid clinicians, albeit with low sensitivity, in identifying early mucosal changes, require trained physicians and dentists to infer the results^11,13,23^. Integration of AI has led to an improvement in accuracy of these devices. AI-based analysis of AFI improved detection accuracy (sensitivity: 80–100%; specificity: 80–91%)^19–21^, while studies have reported that CNN-based diagnosis improves detection of oral cancer using WLI^24,25^. In our study, wherein the dual-mode imaging (AFI and WLI) was integrated with cloud-based CNN, a high sensitivity (87%) and specificity (86%) was achieved, indicating it to be a reliable screening tool. The integrated imaging device allows for automation at three levels with increasing accuracy (Fig. 3). The phone-based neural network (MobileNet), allows a real-time diagnosis of OPMDs/oral cancer (Sensitivity: 79%; Specificity: 82%) and can aid in improving early cancer detection during screening and surveillance. Secondly, enabling the cloud-based Bayesian method further increased the sensitivity of the device to 87%. Finally, a remote telediagnosis by specialists in cases with uncertainty, will ensure accurate triaging of the subject and appropriate referral to a tertiary cancer centre.

In this study, 84% of the patients were evaluated independently by FHWs empowered with dual-mode imaging device, emphasizing the utility of the device in primary health centers lacking specialist expertise. The constant monitoring of the FHWs in terms of imaging and usage of the system enhanced their skills and efficiency. In contrast to the present study, previous studies reported diagnostic adjuncts/devices or assays tested by trained clinicians in hospital settings^13^. Field testing of the device in our study reports significant empowerment of the FHWs in detecting suspicious lesions and an improvement in the overall efficiency of screening (r = 0.8) in low-resource settings. It is to be noted that though the device with an automated algorithm could reliably detect oral neoplastic lesions, trained onsite FHWs are essential to counsel and refer high-risk individuals and to ensure compliance. The findings from this study hence suggests a pipeline that can be adopted at a community level for oral cancer screening/diagnosis; employing FHWs for primary screening and engaging remote specialists in cases of uncertainty (Fig. 3).

The key limitation of the study was the availability of onsite specialist diagnosis only in 16% of the patients. Nevertheless, this cohort of 752 subjects, revealed a good consensus between both the onsite and remote specialist diagnosis (kappa = 0.79). Further, comparison with histology diagnosis showed high sensitivity but low specificity, primarily attributed to the lesser number of patients with non-suspicious lesions who underwent biopsy. The low compliance of the OPMD subjects to undergo biopsy is a significant challenge that needs to be addressed in large-scale screening studies. Histology is the gold standard for the detection and diagnosis of OPMD, however, for practical reasons, WHO has defined Oral Leukoplakia, the primary OPMD, as “a predominantly white patch or plaque that cannot be characterized clinically or pathologically as any other disorder...” To improve the clinical diagnostic accuracy of the definition, several authors have proposed Certainty (C) factors^26^, which defines the parameters for clinical and pathological diagnosis. These factors include, evidence from a single clinical examination (C1; provisional clinical diagnosis), evidence obtained by a negative result of elimination of suspected etiologic factors such as mechanical irritation during follow-up period of 6 weeks (C2; definitive clinical diagnosis), supplement C2 with incisional biopsy (C3; histopathologic diagnosis) and evidence following surgical excision and pathologic evaluation of the excised specimen (C4). In our study, given the objective of establishing a tool to empower FHWs for large scale screening in the field, onsite or remote clinical diagnosis was considered as the gold standard.

This study validated a simple, easy-to-use, economical, and portable PoC diagnostic tool that enables automated diagnosis in low-resource settings. Although continuous monitoring and constant knowledge retention of FHWs improves the efficacy of the screening program, the accuracy of FHW to independently detect lesion was still low, emphasising the need for a diagnostic adjunct. The most significant component of this study is the device being amenable to use by FHWs in low-resource settings for screening and surveillance of oral cancer. With the widespread application of the integrated imaging system validated in this study, it will mitigate the need for an onsite specialist during screening and increase compliance in FHW-led oral cancer screening programs. Adaptation of the results in population-wide cancer screening and early detection programs would enable optimal utilization of human resources in low-resource settings and help in achieving the goal of down-staging oral cancer at diagnosis.

## Methods

### Study design and population

**Figure 4:**
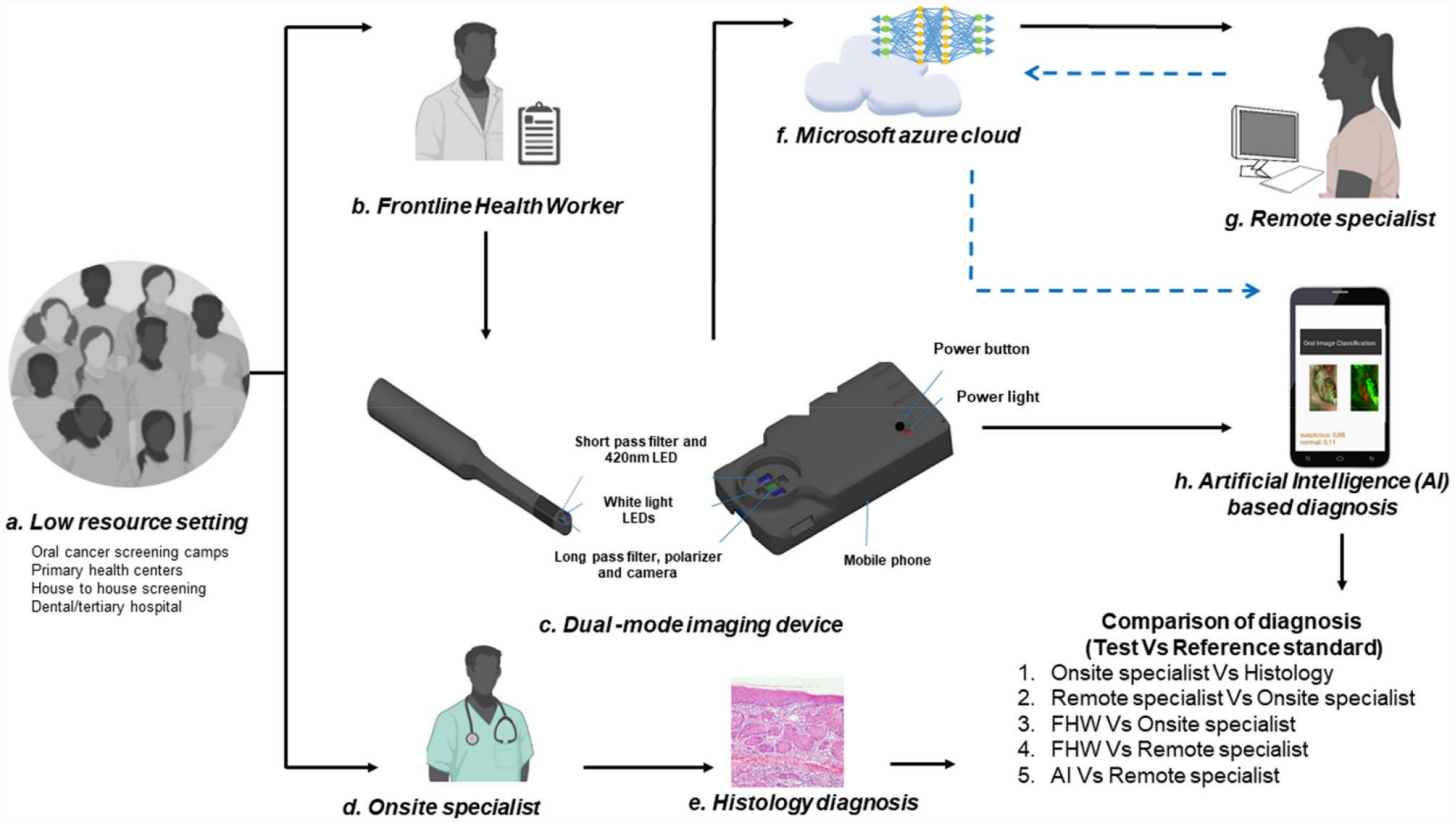
Study design. The study participants were recruited from the tertiary cancer center, dental hospitals, low resource settings like primary health centers, and community camps by Front-Line-Health-Workers (FHWs) (**a**, **b**). The clinical history and images were recorded using the dual-mode imaging device (**c**). FHWs have undergone training for using the dual-mode imaging device and also for identifying Oral potentially malignant (OPMD) and malignant lesions. FHWs diagnosed oral lesions as suspicious if it is OPMD or malignant lesions by direct visual examination. The subjects in dental and tertiary hospitals were re-examined by an onsite specialist by direct visual examination (**d**) and recommended for biopsy (**e**) when required. The onsite specialist diagnosis was compared with histology (reference standard). The images captured by FHWs were uploaded to Microsoft Azure cloud (**f**) and interpreted by a remote specialist (**g**). The tele-diagnosis of remote specialists was compared with the onsite specialist diagnosis (reference standard). The probe images were used for the development of multiple deep learning neural networks (**f**) compared with remote specialist’s diagnosis as the reference standard (**g**). The neural network was integrated with mobile phones (**h**) for testing artificial intelligence-based (AI) diagnosis. The FHW’s and AI diagnosis were compared with the remote specialist (reference standard). (*FHW* Front-Line-Health-Worker, *AI* artificial intelligence). Images (**a**, **b**, **d**, **g**) were created with “BioRender.com” (https://biorender.com), Image (**c**), “The 3D model of the device” was created by solidworks 2020. Image (**f**) was created with Microsoft Paint Windows, along with the original images (**e**, **h**) of the study.

**Figure 5:**
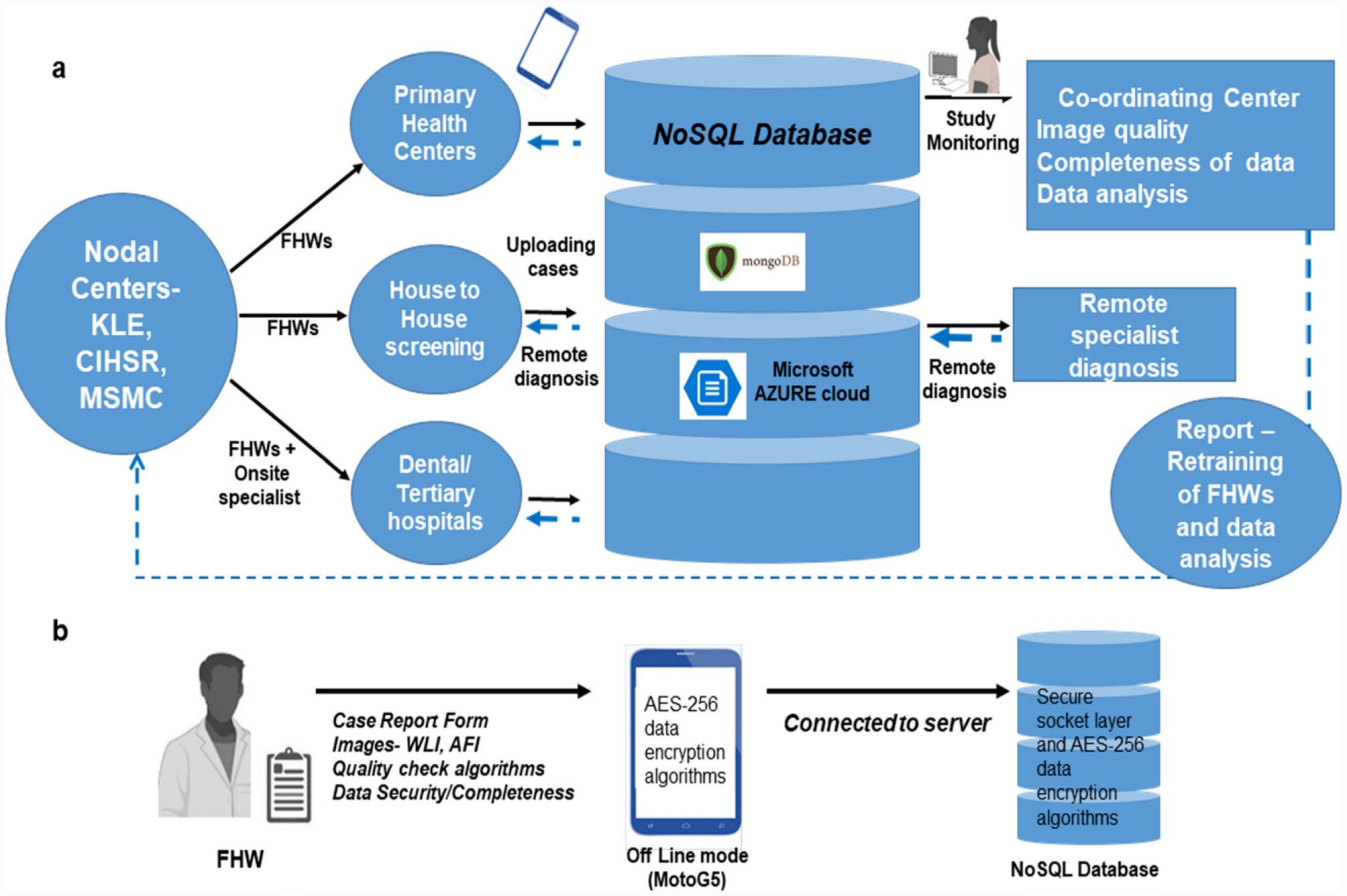
Hub-and-spoke model for data collection. The study nodal centers enrolled FHWs from different study populations (**a**) and participants were recruited according to inclusion and exclusion criteria. The demographics, clinical history of habits/lesions, and images were recorded using a dual-mode imaging device. Intraoral images were captured in the WLI and AFI dual-mode, a large view of the lesion was captured by phone camera, and a more focused image using probe camera by FHW (**a**). The case report form and images were uploaded by trained FHWs to Microsoft Azure Cloud (**b**). The data was stored in a NoSQL database (MongoDB) and was assessed by the study coordinator and remote specialist using the graphic user interface. The interpretation of the remote specialist was sent back to the phone operated by FHW (blue line showing reverse data flow). The study coordinator checked the completeness of data and a specialist checked the quality of images and feedback was sent to respective nodal centers bi-weekly. The data was recorded offline and an AES-256 data encryption algorithm was used to store the data in offline (**b**). *FHW* Front-Line-Health Worker, *WLI* White Light Imaging, *AFI* Auto-fluorescence Imaging, *KLE*, CIHSR, and MSMC: nodal centers. Images of FHW, Study monitoring were created with “BioRender.com” (https://biorender.com).

This is a prospective multi-centre, observational study with a dual-modality imaging device deployed by FHWs in hospital, community, and workplace screening. The study followed the International Conference of Harmonization recommendation on Good Clinical Practice and all methods were carried out in accordance with relevant guidelines and regulations. The study protocol was registered in the Clinical Trial Registry of the Indian Council of Medical Research (CTRI/2019/11/022167, Registered on: 27/11/2019). The subjects were recruited at the study sub-centers, which were monitored by nodal centers in a hub-and-spoke model (Figs. 4, 5). Institutional Ethics Committee approvals were obtained from the three nodal centers- The Karnataka Lingayat Education (KLE) Society’s Institute of Dental Sciences (KLE; ECR/887/Inst/KA/2016), Bengaluru, India, Christian Institute of Health Sciences and Research (CIHSR; EC/NEW/INST/2020/782), Dimapur, Nagaland, India, and Mazumdar Shaw Medical Center (MSMC; NNH/MEC-CL-2016-394), Bengaluru, India prior to the initiation of the clinical trial. The participants who were above 18 years of age, with a history of tobacco smoking and/or chewing or with any oral lesion were included and written informed consent was obtained from all the participants. The individuals currently undergoing treatment for malignancy, pregnancy, tuberculosis, or suffering from any acute illness were excluded.

### Dual mode imaging device

**Figure 6:**
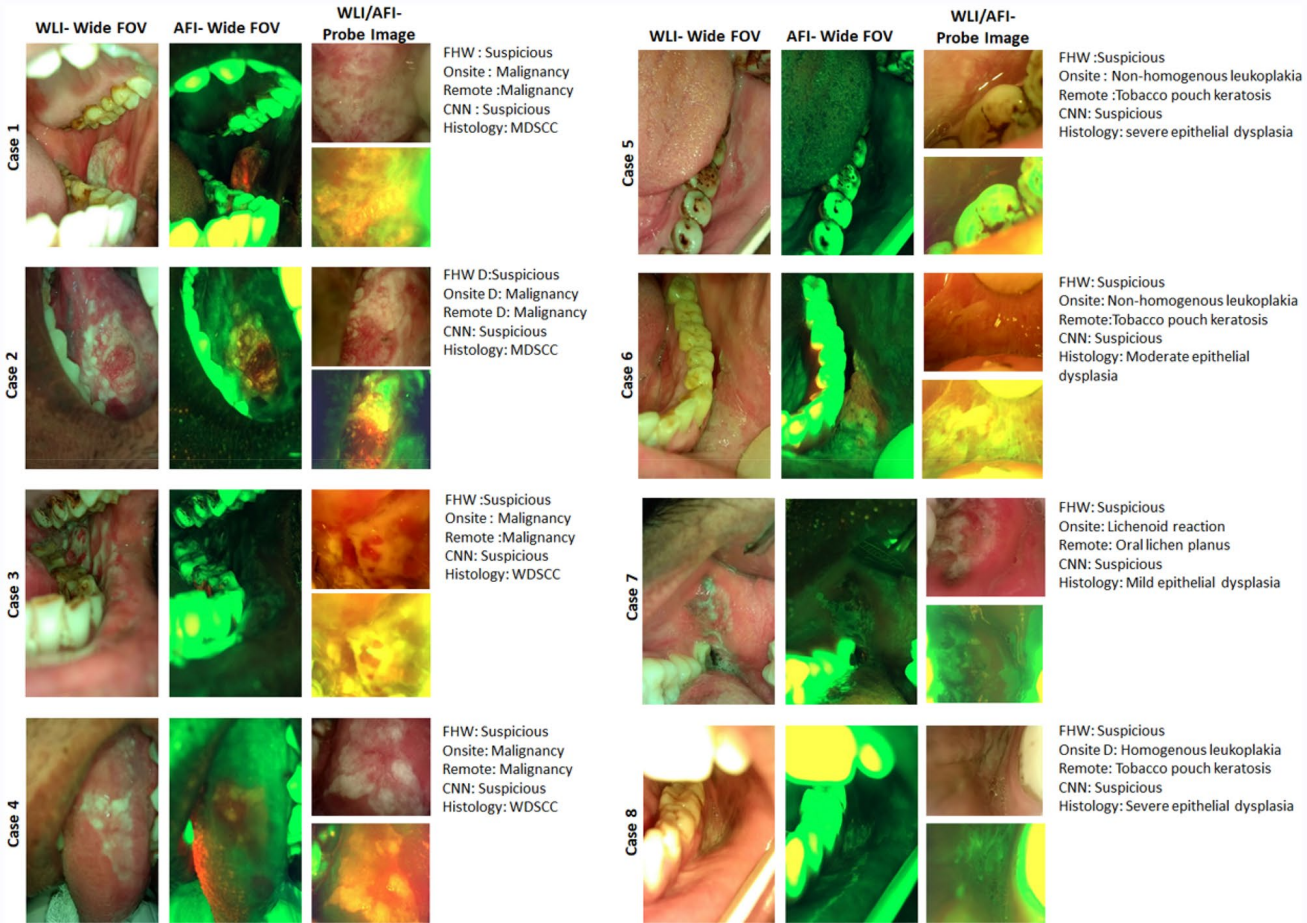
Dual-mode images and diagnosis. The dual-mode imaging device recorded WLI/AFI of the wide field of view (FOV) using a phone camera and focused probe image. The figures depict the diagnosis of FHWs, onsite specialists, remote specialists, CNN (MobileNet), and histology diagnosis of cases. *WLI* White Light Imaging, *AFI* Auto-fluorescence Imaging, *FHW* Front-Line-Health Worker, *WDSCC* Well-differentiated Squamous Cell Carcinoma, *MDSCC* moderately differentiated squamous cell carcinoma, *CNN* Convolutional Neural Network.

The oral cancer diagnostic device used in the study was equipped with dual imaging modalities, comprising WLI and AFI, (405 nm) for both the wide field of view (FOV) based capture of the lesion and surrounding tissues and the intra-oral probe enabling a focused view of the lesion (Fig. 6, Supplementary Figs. S6, S7)^27^.The image capture protocol was carried out as previously reported^16^. Briefly, the smartphone (Moto G5) has a built-in camera, an application for image capture and analysis. The adaptable probe consisted of intra-oral imaging optics for the capture of high-resolution images. The LEDs and drivers for illumination, filters for reflectance and fluorescence imaging, light sensors were incorporated in the mechanical case. The device attached to the Wi-Fi enabled android smartphone was capable of real-time or near real-time synchronization with the server and offline image recording (Fig. 5).

### Training and monitoring of FHWs

The study personnel consisted of FHWs (n = 18), remote specialists (n = 4), onsite specialists (n = 3) and research coordinators (n = 3) (Fig. 5). Specialists provided periodic training (once in three months) to the FHWs on usage of the device and clinical examination of the oral cavity and diagnosis of OPMD, early and advanced oral cancer. In patients with a tobacco-chewing history, wherein visible oral lesions were absent, the FHWs were trained to image the quid-placing site along with the normal appearing buccal mucosa and tongue.

The FHWs worked as a pair and each team were allotted a unique username that was used throughout the study (Fig. 5a). FHWs provided the diagnosis as suspicious (OPMD and/or malignant lesions) or as non-suspicious (other oral lesions) after the direct visual examination. The educational background of the FHWs (non-medical, nursing, dentist, technicians) was recorded (Supplementary Fig. S8). A multivariate analysis was performed on FHW’s experience in the medical field/health programs/current projects, age, and the number of patients recruited to find significant covariates related to the efficiency of FHWs in the detection of suspicious lesions.

### Diagnostic workflow and data flow of the study

All subjects were screened and recruited by the FHWs based on the inclusion and exclusion criteria. The demographic details were collected (Supplementary Fig. S9). The subjects were imaged using the screening device (Fig. 4, Supplementary Fig. S6), and image quality was assessed in real-time by an inbuilt algorithm, which uses an image gradient to measure sharper edges. In subjects with multiple oral lesions, the most suspicious lesion was considered as the index lesion. The FHWs were requested to re-capture if the images appear blurred. The FHWs opined each subject as either suspicious or non-suspicious by direct visualization.

The screened population consists of two clusters based on the involvement of the onsite specialist. One cluster was screened by a team of FHWs with an onsite specialist and the other without an onsite specialist. The subjects in the cluster with the onsite specialist were re-examined by direct visual examination by the onsite specialist, who provided an independent clinical diagnosis.

The data captured (Supplementary Table S3) was blinded and uploaded to a secure server for remote specialist interpretation (suspicious, non-suspicious, or not interpretable) (Fig. 5) based on the wide FOV (both WLI and AFI). The AFI images were interpreted as a gain, loss, or normal fluorescence. The data was encrypted in the phone and the cloud using the AES-256 algorithm without any data loss or compression, using NoSQL-based MongoDB database in Microsoft Azure Cloud (Fig. 5). The recommendation by a remote specialist for further evaluation was communicated to the respective devices. These subjects were evaluated by onsite specialists. Those who are indicated for biopsy by the onsite specialists were referred to tertiary care facilities for biopsy. The research coordinators monitored the quality of clinical workflow, which included number of cases uploaded, data loss, while the image quality was assessed by the specialists from nodal centres. The feedback regarding image quality was given to FHWs and training was repeated whenever required (Fig. 5). The quality of images was also evaluated using Natural Image Quality Evaluator (NIQE)^28^.

The analytical pipeline aimed at comparing the diagnoses obtained in the study to evaluate the efficacy of the personnel and device at different levels. As an initial step, in a sub-cohort wherein onsite diagnosis by the specialist and referral biopsies were carried out to determine the efficacy of the onsite specialist in the delineation of OPMD or oral cancer. Herein histology was used as reference standard. As a next step, to establish the efficacy of the device as a telediagnosis tool, the remote specialist diagnosis was evaluated with onsite specialist diagnosis as the reference standard. Finally, the efficacy of FHW interpretation was compared to the onsite and remote specialist diagnosis.

### Image analysis and pre-processing

We created a combinatorial three-channel data set that combines the information of AFI and WLI of the probe images for analysis^16^. An adaptive histogram equalization method was used to improve the brightness and contrast. The blue channel of the WLI was excluded due to the presence of a long-pass filter in front of the CMOS sensor that blocks the 405 nm excitation light^16^. The green and red channels of WLI and its normalized ratio of AFI were fused to feed to the different pre-trained Convolution Neural Network (CNN) (Fig. 2a)^16^.

### Integration of the convolution neural network (CNN) with the device

The intraoral image dataset used was unbalanced in terms of clinical parameters (site and diagnosis) (Supplementary Table S4). We, hence applied both data and algorithm-level approaches to reduce the influence of the unbalanced dataset by data augmentation and adopted focal loss. First, we built a MobileNet^29^ model (pre-trained with Imagenet dataset, learning rate = 0.0001, batch-size = 32, and epochs = 300), which can be implemented on a smartphone device in real-time (Fig. 2b). We used Nvidia 1080Ti GPU to train the model, which was then converted to TensorFlow Lite format using filter converter (reduces the file size, 16.3 MB). The customized android smartphone application to control the screening device was also implemented with MobileNet based classification approach. The user could employ the android application to analyze the captured images using the proposed CNN model without an internet connection. The moto G5 android smartphone (with octa-core 1.4 GHz CPU, Adreno 505 GPU, and 2 GB RAM) took either 306 ms (CPU) or 288 ms (GPU) to process image pairs.

We developed another CNN based on the Bayesian deep learning (BDL)^30^ framework (Fig. 2c), which provided a prediction and an uncertainty value (implemented on the cloud server). We used the Monte Carlo Dropout Network (MCDN)^31^ to obtain the model uncertainty, which can be interpreted as a Bayesian approximation of a Gaussian process. We trained the BDL with the dual-modal intraoral images using VGG19 (epochs = 300, learning rate = 0.0001, decay 5 times by every 20 epochs, batch size = 32). Two dropout layers with 0.5 rate were applied to the last two fully connected layers to implement the MCDN. The Bayesian deep network model was implemented on the cloud server, which could produce predictions as well as correlated uncertainty value.

### Statistical analysis

The sample size for validation was calculated with 80% power to detect a non-inferiority difference of 0.10 using a one-sided binomial test with a significance level of 0.05 (sensitivity with the mobile intra-oral screening device is > 86.5%). The sensitivity, specificity, Negative Predictive Value (NPV), and Positive Predictive Value (PPV) of different screening methods were calculated. The agreement between diagnoses was examined using Kappa statistics. F1 score was calculated to compare the diagnostic efficiency of FHW. Multivariate analysis was performed using linear regression to assess the effect of categorical and numerical covariates on the relevant outcome. P-value less than 0.05 was considered. The F1 score was calculated as below:

$${\text{Precision}} = {\text{True}} \, {\text{Positive}} / ({\text{True}} \, {\text{ Positive}} +{\text{False}} \, {\text{Positive}})$$

$${\text{Recall}} = {\text{True}} \, {\text{Positive}} / ({\text{True}} \, {\text{ Positive}} +{\text{False}} \, {\text{ Negative}})$$

$${\text{F1Score}} = 2 * ({\text{Precision}}* {\text{Recall}})/ ({\text{Recall}} + {\text{Precision}})$$

## Supplementary Information

Supplementary Information.

## Data Availability

All data generated in this study is provided in the manuscript is available at https://www.msctr.org/wp-content/uploads/2021/09/Field-validation-of-ANN-based-PoC-device.zip. The code is available at https://github.com/ocscode/ocs_project. Additional, de-identified image data can be made available to other researchers in the field upon request proposals and approval by the study management committee. Requests should be directed to the corresponding author/s.
