# Supplementary material for: Field validation of deep learning based Point-of-Care device for early detection of oral malignant and potentially malignant disorders
Source: Sci Rep. 2022 Aug 22;12:14283. doi: 10.1038/s41598-022-18249-x (PMC9395355; doi:10.1038/s41598-022-18249-x)
Supplement: Supplementary file 1 — Supplementary Information. [file 41598_2022_18249_MOESM1_ESM.pdf]

Title :

Field validation of deep learning based Point-of-Care device for early detection of oral malignant and potentially malignant disorders

Praveen Birur N[1,2,3Ŧ], Bofan Song [4 Ŧ], Sumsum P Sunny[2,5 Ŧ], Keerthi G[1], Pramila Mendonca[5], Nirza Mukhia[1], Shaobai Li[4], Sanjana Patrick[3], Shubha G[1], Subhashini AR[1], Tsusennaro Imchen[6], Shirley T Leivon[6], Trupti Kolur[5], Vivek Shetty[5], Vidya Bhushan R[5], Daksha Vaibhavi[1], Surya Rajeev[1], Sneha Pednekar[1], Ankita Dutta Banik[1], Rohan Michael Ramesh[6], Vijay Pillai[5], Kathryn OS[7], Petra Wilder Smith[7], Alben Sigamani[8] Amritha Suresh[2*], Rongguang Liang[4*], Moni A Kuriakose[2,5*]
Ŧ Equal contributions
*Corresponding authors

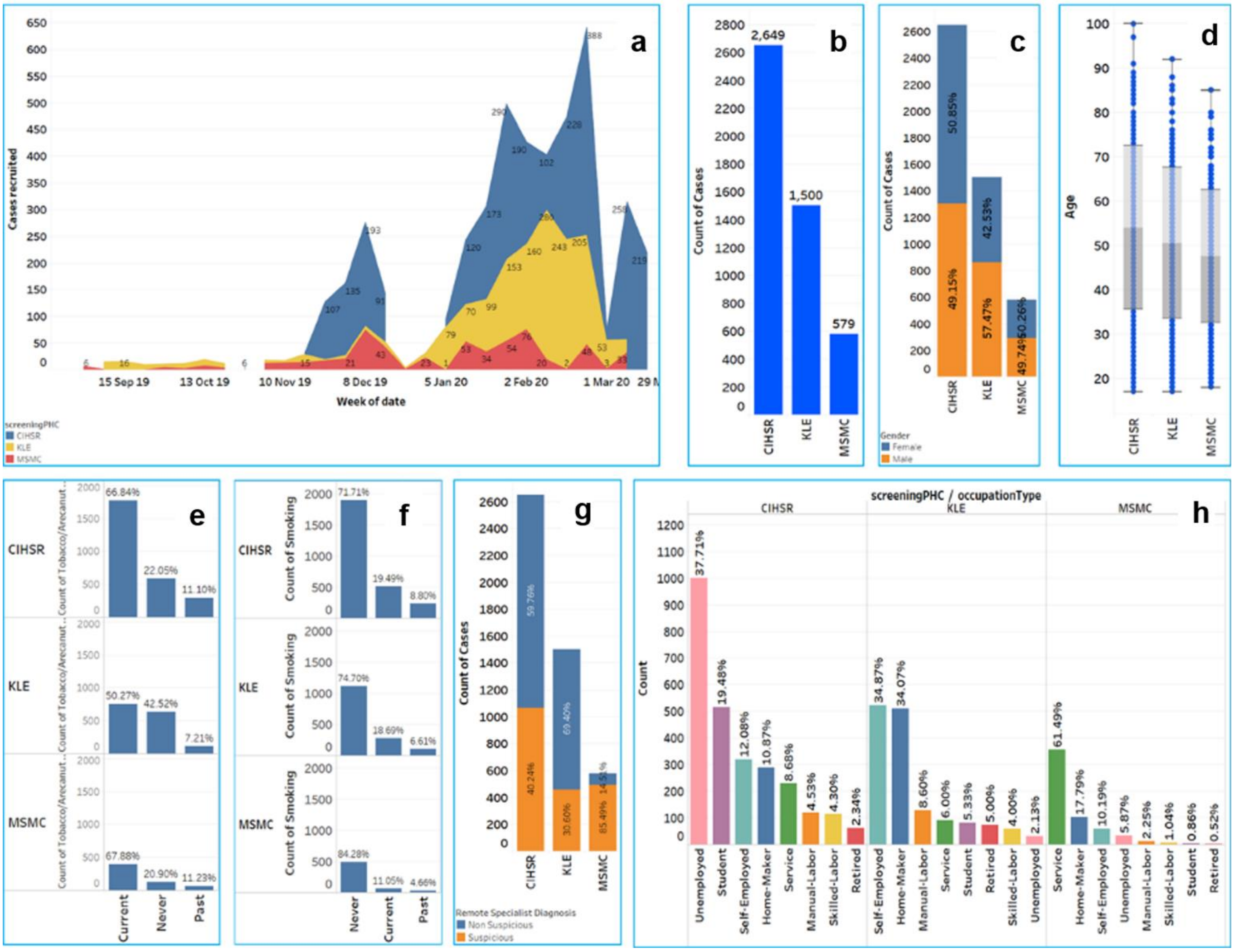

Figure S1. Demographics of study Participants

Distribution of the number of subjects, gender, and age in different centers (a-d). The habit of tobacco chewing (e) and smoking (f) profile of patients in each center. The percentage of suspicious lesion(g) and occupation of subjects in each center (h). (Study centers- KLE, MSMC, CIHSR)

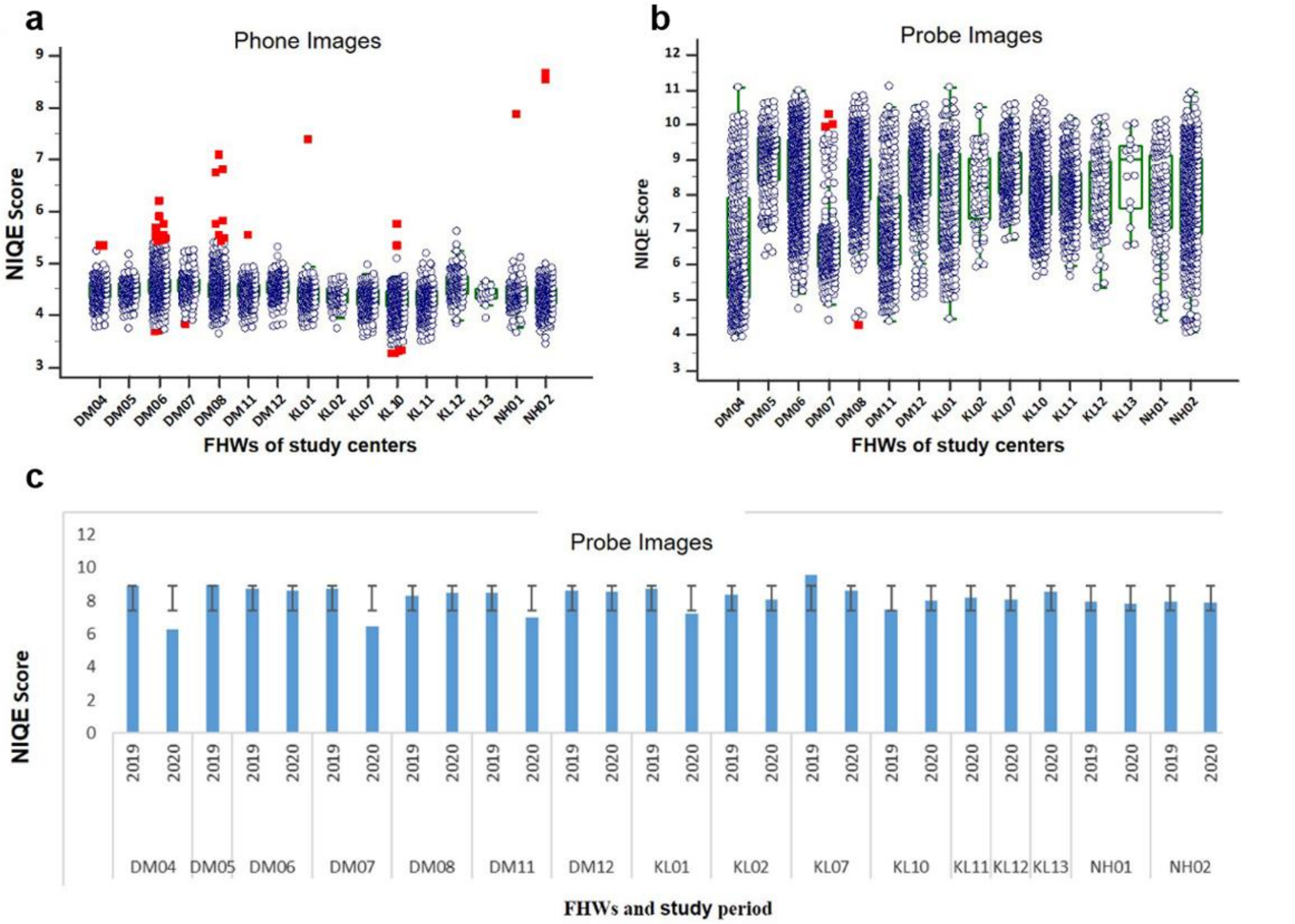

Figure S2. Quality of Images

Natural Image Quality Evaluator **(**NIQE) score of phone (a) and probe images (b) of each FHWs and the improvement in quality of probe images in the second quadrant of study (c) (Mean ± Standard deviation).

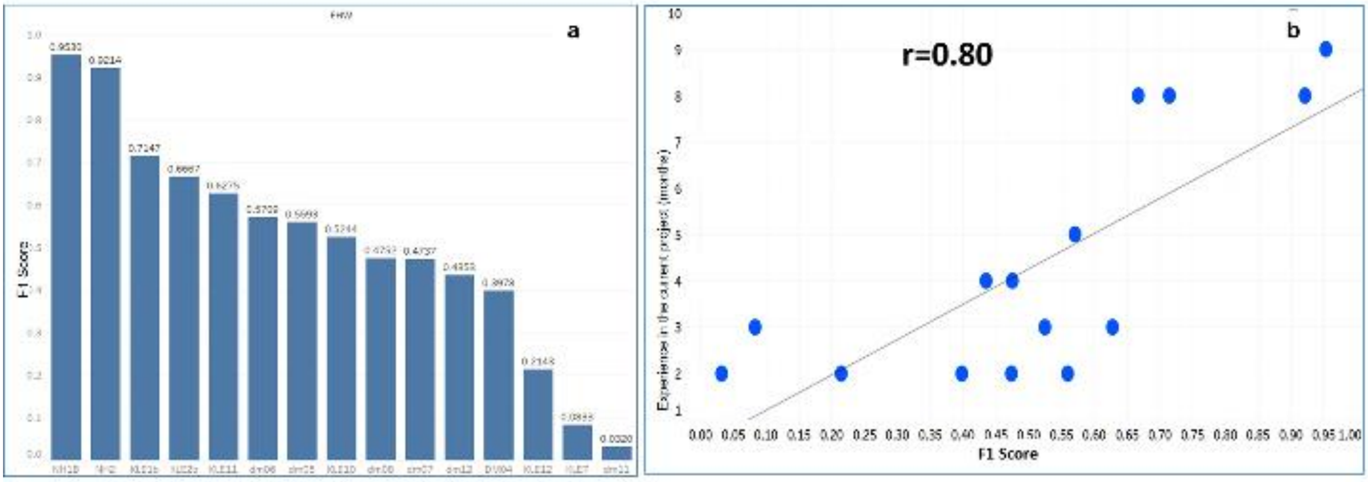

Figure S3. F1 Score of FHW and correlation with experience in current project

F1 score of each FHW (a) and correlation of their experience in current project to F1 Score (b).

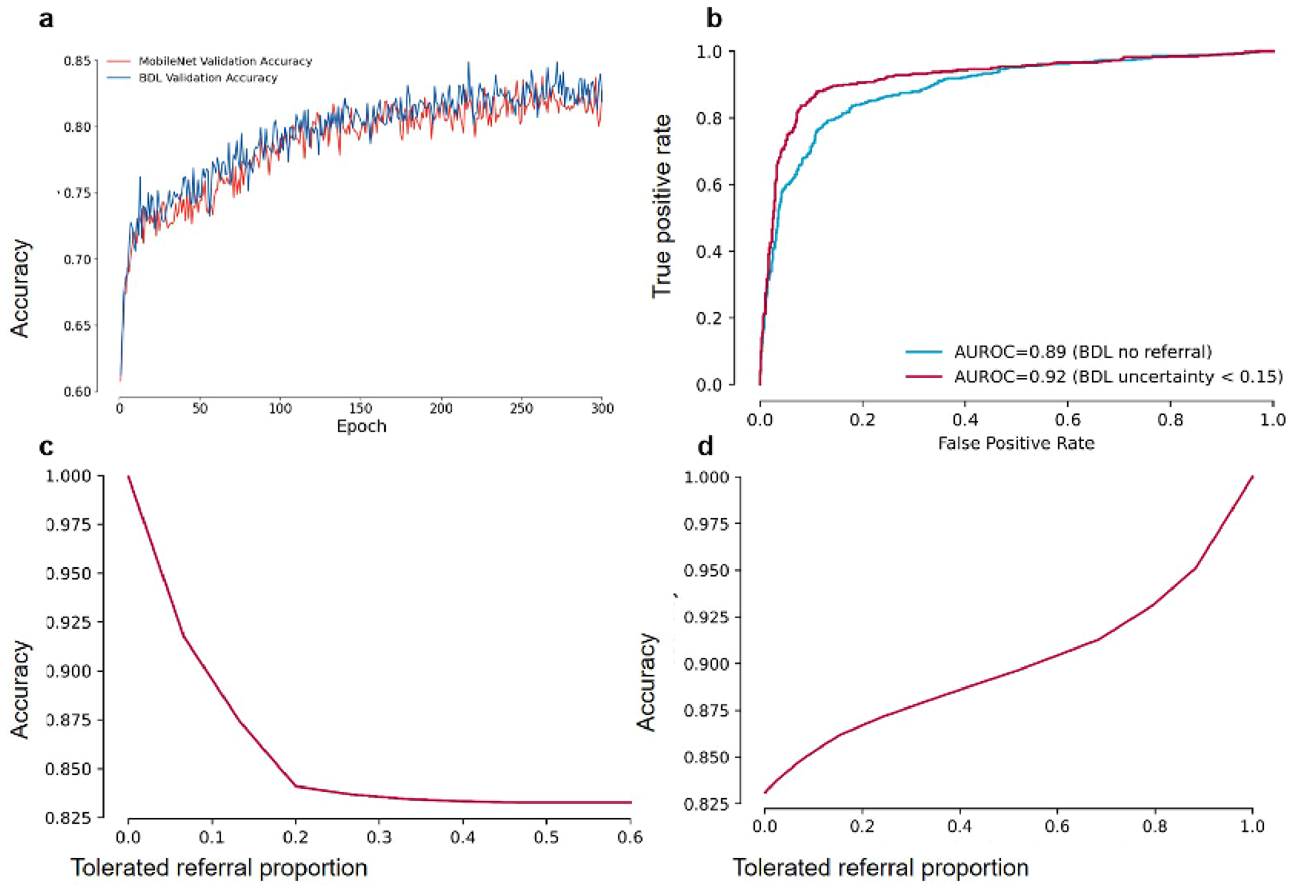

Figure S4.Convolutional Neural Network validation

The validation accuracy of MobileNet and Bayesian deep learning framework (a) in delineating suspicious and non-suspicious lesions. The Bayesian deep learning(b) with referral uncertainty value showed the area under the curve of 0.92 in the validation stand-alone dataset. The change of standalone dataset accuracy correlates to the tolerated model uncertainty value and referral proportion (c,d) by BDL. (BDL: Bayesian deep learning, AUC:Area Under Curve).

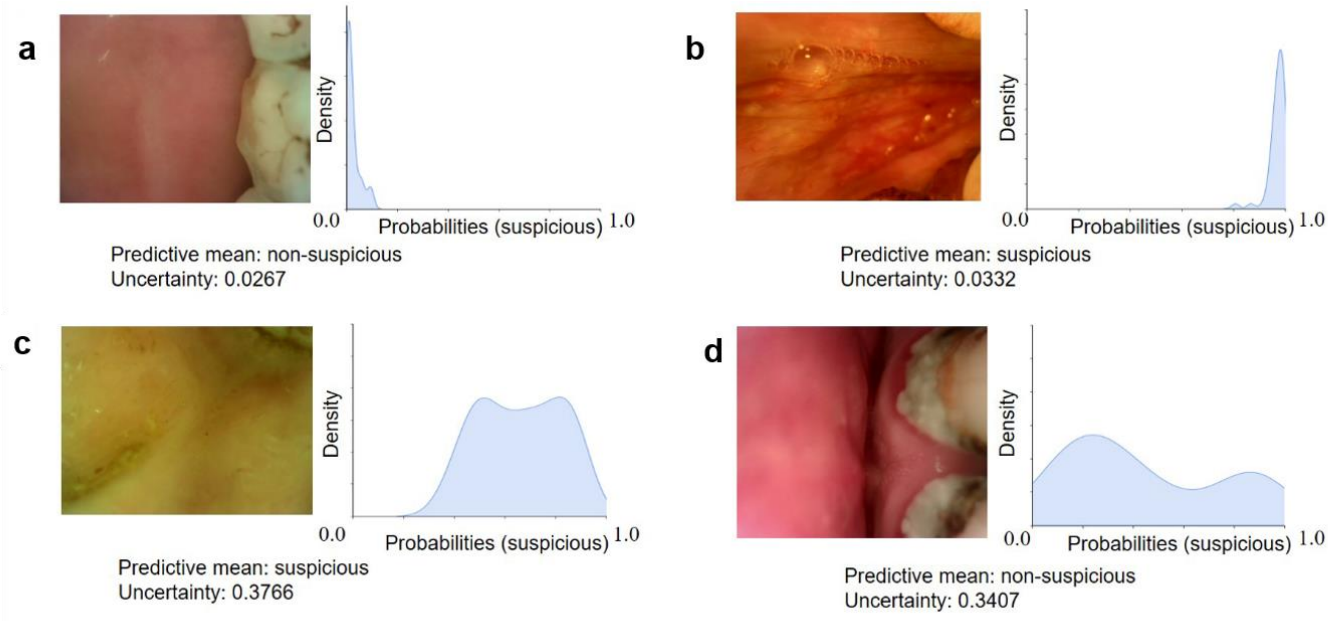

Figure S5. Uncertainty prediction of images

The intraoral images and corresponding approximate predictive probability posteriors distribution produced by the Bayesian neural network. (a) confidently classified as non-suspicious, the sampled probability (suspicious) predictions are near zero (b) confidently classified as suspicious, the sampled probability (suspicious) predictions are near 1.0. (c,d) uncertain by the Bayesian neural network with the wider posterior distribution.

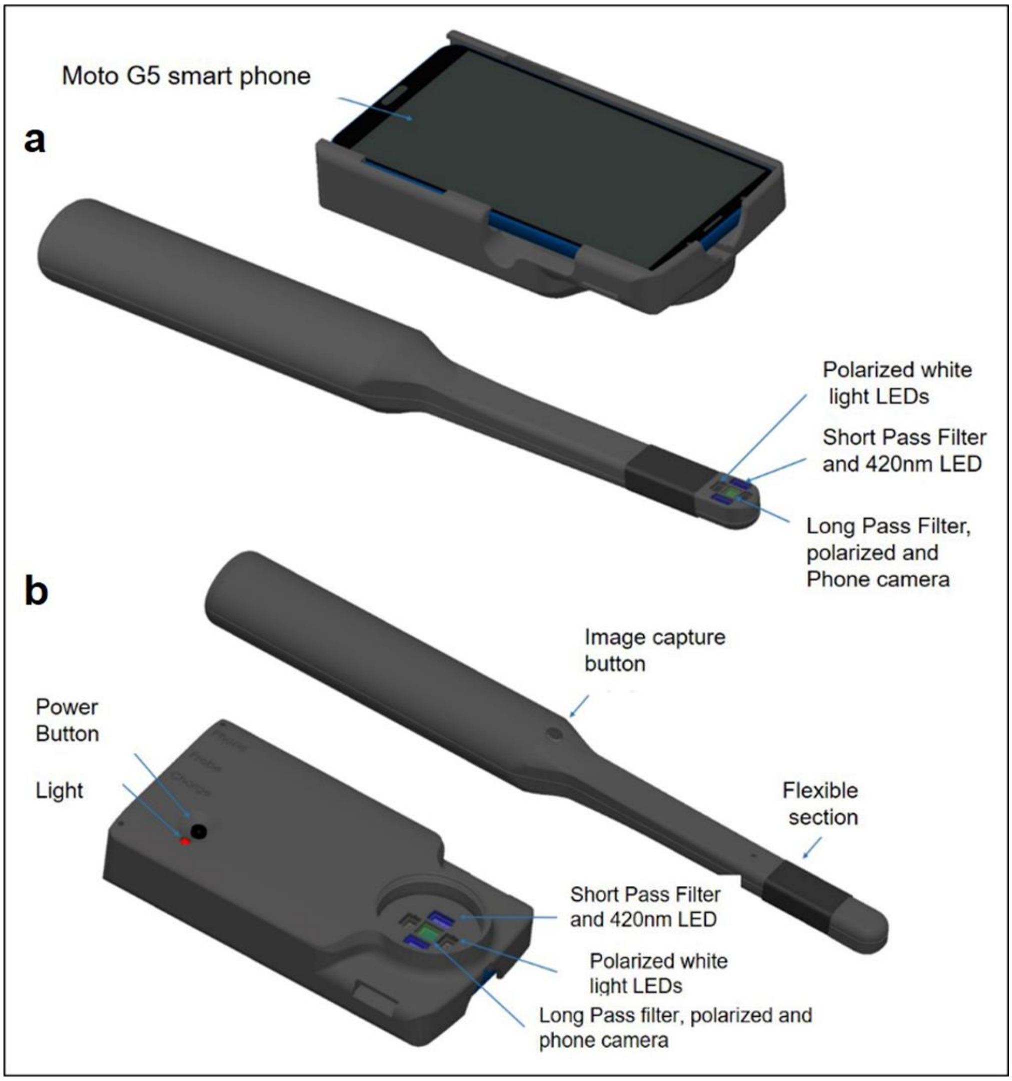

Figure S6. Dual Modality Screening device

The smartphone-based dual-mode oral cancer screening system rendered images (a,b).

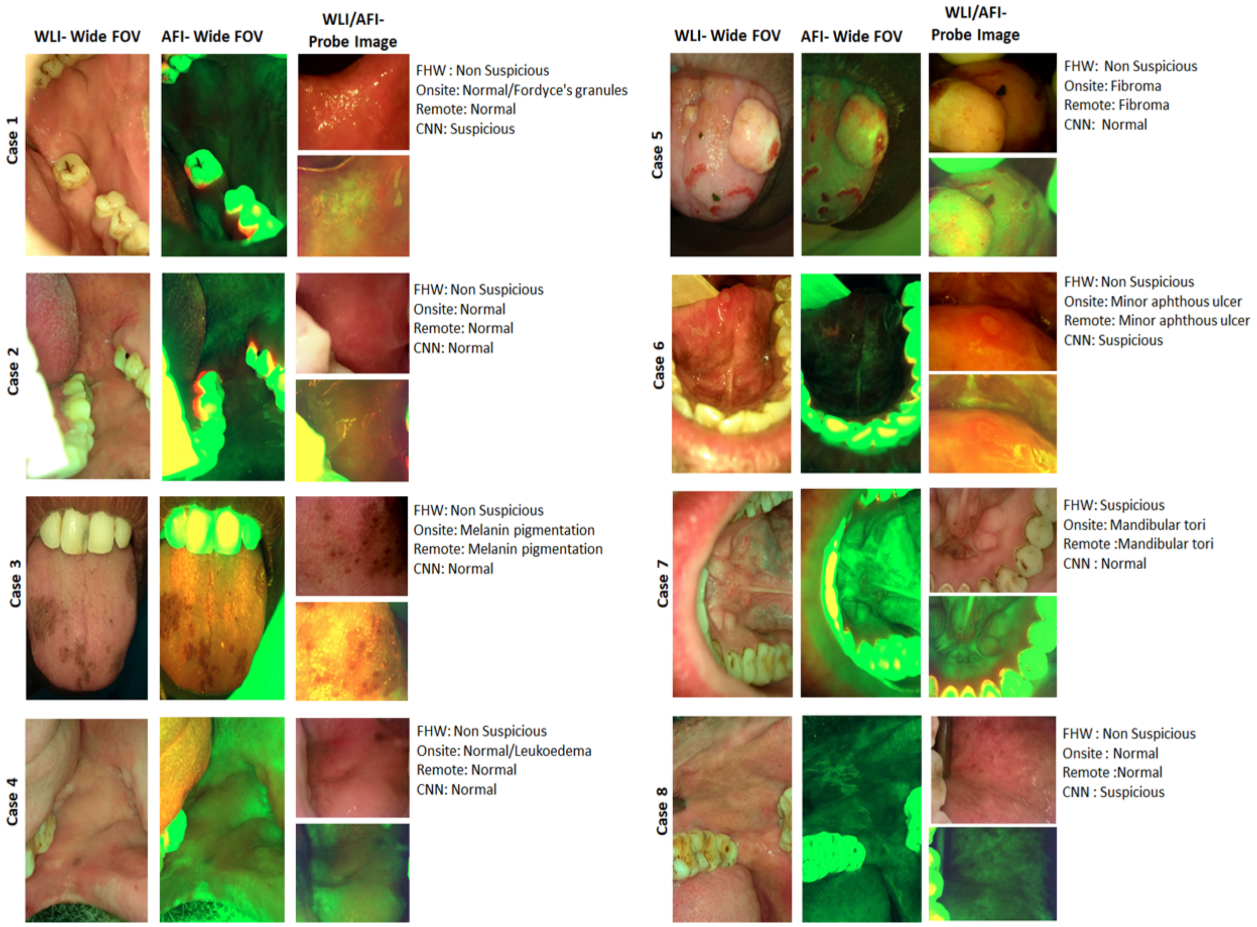

Figure S7. WLI and AFI of wide FOV and probe images

Images of Non-suspicious cases and diagnosis of FHW, onsite specialist, remote specialist, CNN, and histology diagnosis (WLI: White light imaging; AFI: Auto-fluorescence Imaging; FHWs: Frontline-Health-Workers; FOV: Field of view)

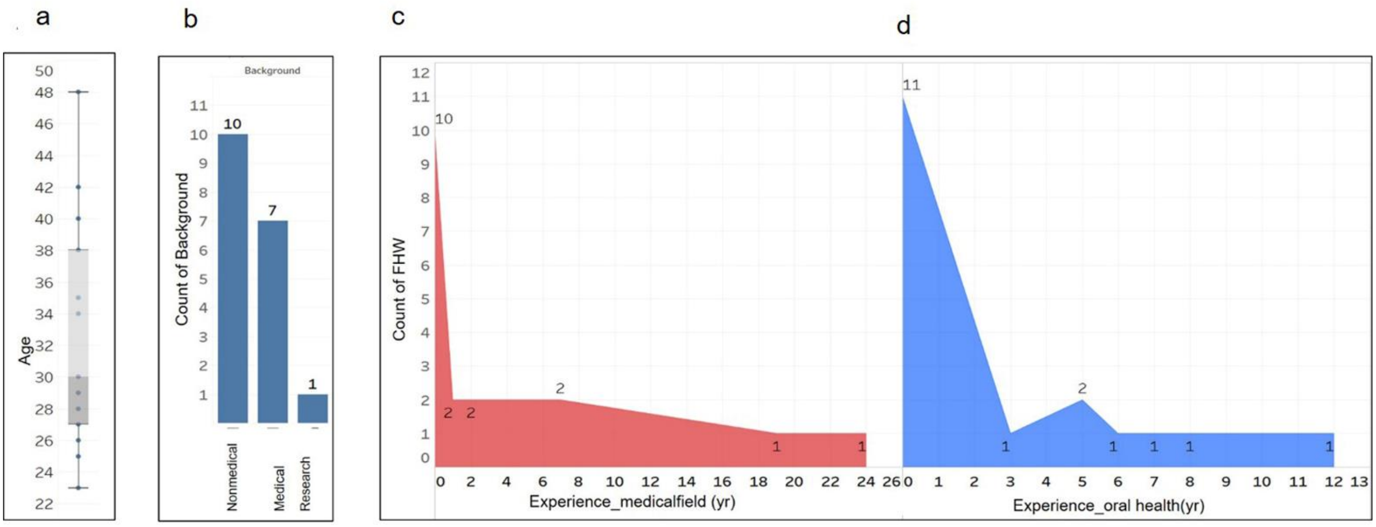

Figure S8. Profile of FHW

The distribution of age (a), number of FHWs from the different backgrounds that they worked previously before the study (b), and their experience (c,d) in the specific background in years.

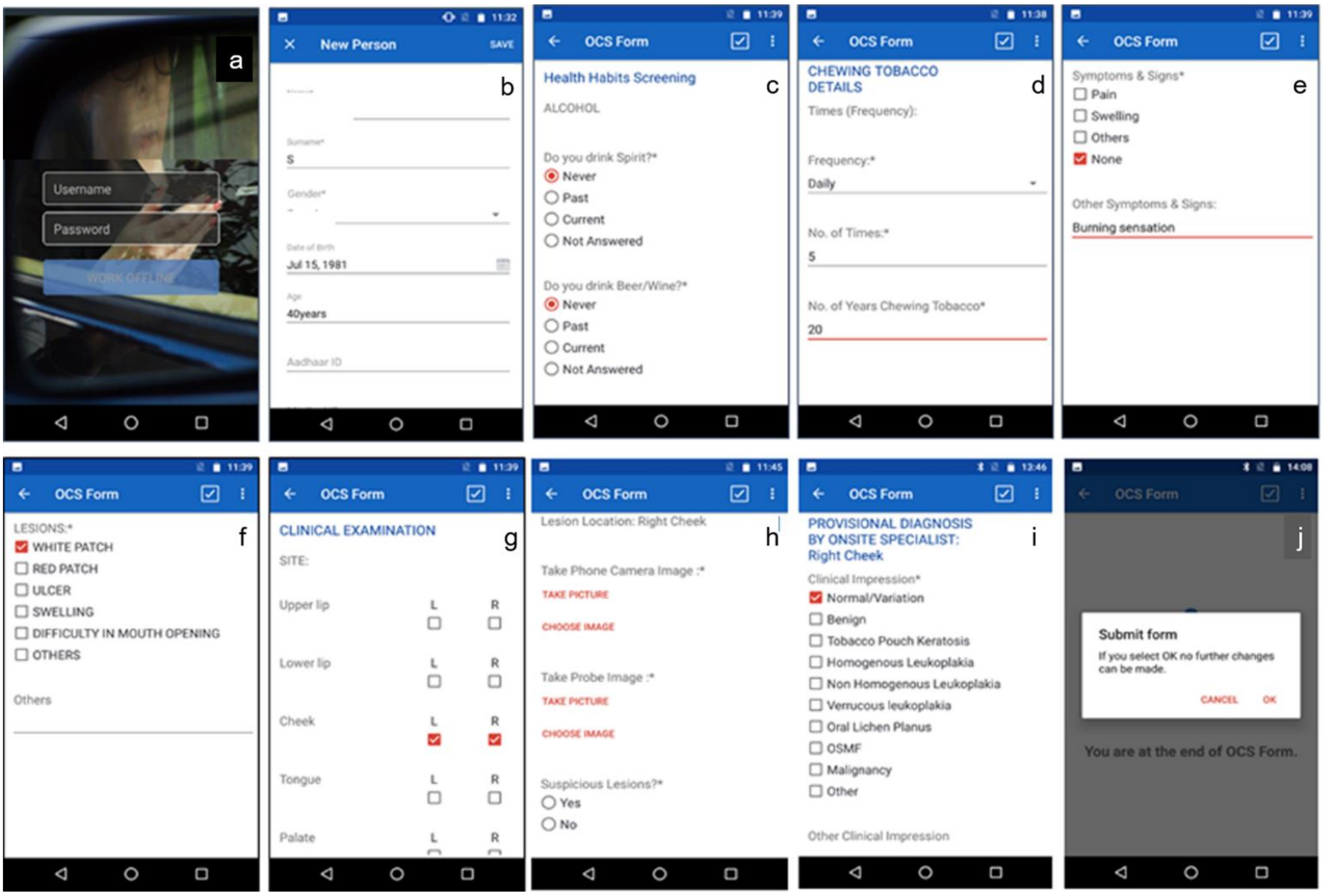

Figure S9. Graphic User Interface of Case Report Form

Represents graphic user interface of case report form used in the mobile phone by FHW (a-j). The FHW logs in with username and password (A) and enters demographic details (b) with tobacco usage (c,d). The clinical findings were recorded (e-g) and images (h). The case report form was submitted after adding the interpretation of FHW (h) and onsite specialist (i-j) diagnosis (if present).

Dependent Y	F1				
Least squares multiple regression					
Method	Enter				
Sample size	18				
Coefficient of determination R^2	0.8665				
R^2-adjusted	0.7163				
Multiple correlation coefficient	0.9309				
Residual standard deviation	0.1414				
Regression Equation					
Independent variables	Coefficient	Std. Error	$r_{partial}$	t	P
(Constant)	0.4659				
Age of FHW	-0.009719	0.00705	-0.4381	-1.379	0.2053
Medical device used previously(in years)	0.09874	0.04435	0.6186	2.227	0.0566
Experience in current projects (months)	0.1044	0.02455	0.8325	4.25	0.0028
Experience in Health programm (years)	0.03907	0.01766	0.6161	2.212	0.0579
Experience in medical field (years)	-0.08373	0.03058	-0.6955	-2.738	0.0255
Experience in oral health programs (years)	-0.1331	0.04952	-0.689	-2.689	0.0276
Experience in using mobile phone (years)	0.0016	0.016	0.03533	0.1	0.9228
Experience in research (years)	0.1055	0.0451	0.6372	2.338	0.0475
Total cases recruited	-0.000219	0.000215	-0.3389	-1.019	0.3381
Analysis of Variance					
Source	DF	Sum of Squares	Mean Square		
Regression	9	1.0377	0.1153		
Residual	8	0.1599	0.01998		
F-ratio	5.7702				
Significance level	P=0.0108				

Table S1.Multivariate analysis of the profile of FHWs with F1 Score

The experience of FHW in the medical field showed significant association with the duration of experience in the current project (F1 Score).

	Model Size	Million Parameters	Accuracy (Standalone test dataset)
**MobileNet**	16MB	4.2	79%
**InceptionV3**	92MB	23.8	81%
**VGG19-BDL (no referral)**	549MB	143.7	83%
**VGG19-BDL (uncertainty<0.15)**	549MB	143.7	87%

Table S2. Neural Network model and accuracy on test data set

The comparison of model size, parameter number and standalone dataset accuracy between MobileNet, InceptionV3 and VGG19.

Number	Contents of case report form
1	Name
2	Gender
3	Date Of Birth/Age
4	Phone Number
5	Address
6	Consent Form Signed
7	Occupation Type
8	Family History Of Cancer
9	Smoking History
10	Chewing Tobacco/Arecanut History
11	Alcohol History
12	Symptoms And Signs
13	Lesions Type
14	Lesion Location
15	FHW Interpretation
16	Onsite Specialist Diagnosis

Table S3. Contents of case report form recorded by FHW

Diagnosis by Remote specialist			Oral sub-sites		
	**Training set**	**Test set**		**Training set**	**Test set**
Normal	3211	700	Buccal mucosa	3950	929
Benign	740	112	Lower vestibule	216	141
Oral lichen Planus	36	13	Tongue	256	82
Oral sub-mucous fibrosis	61	51	Lip	652	165
Tobacco pouch keratosis	672	278	Floor of mouth	11	5
Homogenous leukoplakia	309	164	Palate	84	20
Non-Homogenous leukoplakia	268	47	Retro-molar trigone	31	46
Malignancy	32	51	Other	129	28
Total	5329	1416		5329	1416

Table S4 . Distribution of training and test data-set according to remote diagnosis and site

The image distribution used for CNN training and test showing unbalanced data in different oral sub-sites and oral lesions.

Short write up:

1.Step-wise description of the Work flow of the study:

1.1 Background

Our previous studies have proved that FHWs empowered with mobile health (mHealth) with decision-based algorithms, aid in remote early detection of oral cancer in resource constrained settings. m-Health approach can be a strategic plan to connect specialists and downstage oral cancer in low-resource settings. In addition, the need for a POC diagnostic tool, which is non-invasive, can be used by low skilled healthcare personnel and is cost effective was realised . The present study was undertaken to validate the mHealth based POC tool deployed by Frontline Healthcare Workers (FHWs) in the field, developed for the early detection of oral cancer and oral precancerous lesions and to empower FHWs with diagnostics.

1.2 Approach

The primary aim of this study was to evaluate the efficacy of the Convolutional-Neural-Network (CNN) -enabled device as a Point-of-Care (POC) diagnostic tool for oral cancer screening in low-resource settings. This is a prospective multicentric, observational study, using a smartphone-based dual-view, dual modality imaging device. The dual-view captured images with a wide field of view and intraoral probe view that provides a close focus imaging of the oral cavity and dual-mode comprises of white light (WL) and Autofluorescence Imaging (AFI). Ethical clearance was obtained for the study from all the sub-centres, and was registered under CTRI (Clinical Trial Registration India).

1.3 Description of the oral cancer screening device

The screening equipment consisted of a smartphone and a dual-modality imaging device. The smartphone (Moto G5) comprised of a built-in camera, and an application for image capture/analysis. The dual-mode comprised of white light (WL) and Autofluorescence imaging (AFI- 405nm). Dual-view modality captured images of oral cavity with wide field of view and an intra oral probe view provided a close focus of lesion site. The wide field of view facilitated remote specialist diagnosis, whereas the probe images aided in developing AI algorithms, and closer view of lesion. The adaptable probe made use of intra-oral imaging optics for the capture of high-resolution images. The LEDs and drivers for illumination, filters for reflectance and fluorescence imaging, as well as light sensors were incorporated in the mechanical case. The device attached to the Wi-Fi enabled android smartphone was capable of real-time or near real-time synchronization with the server and offline image recording.

1.4 Healthcare personnel and study sub-centres

The study personnel involved FHWs (n=18), remote specialists (n=4), onsite specialists (n=3) and research coordinators (n=3). The participants were recruited at the study sub-centres, which were monitored by nodal centres. The KLE Society's Institute of Dental Sciences, (KLE) Bengaluru, India, Christian Institute of Health Sciences and Research, Dimapur India, and Mazumdar Shaw Medical Centre (MSMC), Bengaluru, India, served as nodal centres. All the participants who were aged above 18 years and individuals with history of tobacco smoking/chewing, or having any oral lesion and who consented to the study were included. The individuals currently undergoing treatment for malignancy, pregnancy, tuberculosis, or those suffering from any acute illness were excluded.

1.5 Training and monitoring of Healthcare personnel

The specialists trained the healthcare workers for visual oral examination , to use the mobile application and to image oral lesions. Pre and post tests were conducted to assess their knowledge and skills for oral cancer screening. The FHWs performed screening in hospital-based settings (KLE Society's Institute of Dental Sciences and MSMC), house-to-house screening, and in outreach programs. FHWs were instructed to capture images of the suspicious lesions or if lesions were not identified, then normal subsites of the oral cavity were

captured. The FHWs worked in pairs as a team, where each team was allotted a unique user ID and a password, which was used throughout the study. The ID aided in monitoring FHWs during the study for quality of images, and clinical data collection. The trained health care workers screened the participants, obtained the risk-factor data, images of oral lesions and uploaded the data for remote specialist consultation. Based on the specialist recommendation the participants were triaged for further management.
The healthcare workers were trained to use binary classification for diagnosing oral lesions as 'Yes' or 'No' for 'suspicious' and 'non-suspicious lesions' respectively. Following the collection of the required information in the mobile application, FHWs provided their opinion as suspicious or non-suspicious lesion. OPMDs and oral cancer were considered as suspicious oral lesions and benign, normal and normal variations were considered to be non-suspicious. The specialists were also internally trained for standardization of diagnostic terms in the study.

1.6 Evaluation

The total population screened (n=5025 participants with 32,128 images) was divided in two groups for analysis purpose. The hospital- based settings (KLE Society's Institute of Dental Sciences and MSMC), which had Onsite specialist was considered as group 1, whereas group 2 included the rural settings (Dimapur, North East India) , outreach screenings, and house-to-house screening, and had no onsite specialist. Participants were rescreened by onsite specialists following FHW screening whenever a specialist was present. The onsite specialist provided specific clinical diagnosis for both White light Images (WLI) and Autofluorescence Images (AFI). The clinical diagnosis for WLI were tobacco pouch keratosis, Homogenous leukoplakia, non-homogenous leukoplakia, Oral submucous fibrosis, Oral lichen planus, verrucous leukoplakia, benign, normal, and others. AFI were interpreted as loss, gain, normal or not appreciable. All the data captured by FHWs in both the groups, irrespective of the presence or absence of an onsite specialist, was uploaded to a secure server for remote specialist recommendation. The remote specialist then provided their specific diagnosis for WLI, and opined on AFI as similar to onsite specialist for the uploaded data.

1.7 Web portal setup

The super admin from the central team assigned trained specialists for each sub-centre. Each team consisted of a local coordinator, corresponding local trained healthcare workers, and the allocated specialist for the respective sub-centre. There were three teams formed at three sub-centres, and the healthcare workers were under the supervision of the local coordinator of the respective sub-centre. The super admin could assess the de-identified data of all the sub-centres, and constantly reviewed and monitored the number of cases recruited, the number of active FHWs. An alert was raised whenever there were pending cases for remote diagnosis for more than 48hours. The setting up of passwords, creation of usernames, addition or deletion, and activation or deactivation of healthcare workers was controlled by the super admin and was password protected. The dashboard was designed in such a way that the remote specialist and the local coordinator assessed only their allotted subsites. The study was Health Insurance Portability and Accountability Act (HIPAA) compliant, and the super admin, research co-ordinators, local co-ordinators could extract the Comma-separated values (CSV) files for monthly analysis.

The designated remote specialist assigned had an access to the dashboard using a specific username and password, to the assigned sub-centre allotted to them. Hence, they could only view the cases and gave their recommendation on a day-to-day basis. When in doubt, the remote specialist forwarded the case for a second opinion to reach a suitable diagnosis. The specialist who provided a second opinion was an expert in the field of Oral Medicine, with 20 years of experience and also had an exclusive experience in mHealth for about a decade. The remote specialist recommendation was communicated back to the respective devices as a notification message, and patients with suspicious lesions were referred to tertiary care centres for further evaluation and/or biopsy. All participants were advised to quit the habit, and regular follow up care and were referred to tobacco cessation centres.

The total number of cases recruited and enrolled for the study was updated in google form on regular basis. The google form was updated once in two weeks by the local coordinator at the respective sub-centres enrolled for the study, and they constantly monitored FHWs. The local coordinator had access to the identified data, thereby monitoring all the participants who required immediate care. The local coordinator could download the CSV file, and images corresponding to their respective centres.

The research coordinators monitored the quality of the clinical work including the number of cases recorded. They also alerted the FHWs in case images were interchanged for the selected sub site of the oral cavity, for example, if the FHWs had selected right buccal mucosa and captured the left one. They also deleted the incomplete data and duplicate forms created. In addition, the research coordinator downloaded the images every weekend on a regular basis and performed the quality check of the images using the Naturalness Image Quality Evaluator (NIQE) software. The feedback regarding image quality and data capture was provided to the FHWs, and they were retrained as required.

The server had an integrated AI algorithm, thereby providing an automated AI diagnosis for every uploaded case. Both group 1 and group 2 participants were diagnosed by FHWs, Specialists, and AI. To be more specific, FHW opinion had to be given as “yes” or “no” for suspicious oral lesions for group 1 participants. In addition, onsite and remote specialists provided the specific diagnosis and AI diagnosis as mentioned above for the group 1. For Group 2 participants, FHW opinion was provided as either “yes” or “no” for suspicious oral lesions, and remote specialist gave the specific diagnosis similar as above for both WLI and AFI, and automated AI diagnosis was also provided. The biopsy was performed based on the clinical examination by the specialist. The histopathological diagnosis were obtained whenever available, and the excel sheet was updated.

1.8 Diagnostic accuracy

Histopathological diagnosis was considered as the gold standard. The onsite specialist diagnosis was compared to histopathological diagnosis whenever available. Being an invasive procedure, the compliance for biopsy is low specially for asymptomatic lesions in Indian set-up. It was challenging to perform biopsy for all the identified suspicious cases in the field settings, and in outreach activities such as door-to-door screening. Remote specialist diagnosis was compared to the onsite specialist diagnosis, which was considered the reference standard. Our previous studies have proved that the remote specialists are as efficient as the onsite specialists in diagnosing oral lesions (Birur NP et al. IJC;2019). Therefore, the remote specialist diagnosis was then considered as the reference standard, in sub-centres which had no onsite specialists and where histopathological diagnosis was not established. FHW diagnosis and AI diagnosis were compared with the remote specialist diagnosis.

Study challenges and solutions–

There was often a time lag while capturing probe images, and sometimes a black screen would appear, as the phone frequently hanged as these devices were prototypes. In initial days this resulted in longer time to screen. This was the greatest field challenge faced by healthcare workers. This issue was resolved by re-training to handle the devices; ensuring that wires are untangled, ensuring proper connections, and proper focusing of the probe while capturing images could speed up the process, thereby preventing the frequent hanging of the probe.

Another major challenge was the creation of duplicate or multiple case report forms by FHPs. This was a major concern, as only completed cases could be uploaded and be made available for remote specialist diagnosis and consultation. The case report forms in progress remained on the phone, utilizing a large amount of phone storage space. Therefore, constant manual deletion of duplicate or multiple forms was required. This issue highlighted the need for constant reinforcement and re-emphasis on the usage of the application by FHPs. Another issue of concern was that once the patient registration was done, but failed to create the case report form, the registered patient’s name remained on the phone. The technical team had to automatically delete the names of these patients that had no case report forms the following

next day. In the low resource settings, sometimes, owing to poor connectivity, there were issues with uploading images due to a sync error, reattempts were made with good connectivity.
In the initial days of screening, the password expiry notification was not available in advance. This issue had created an inconvenience to access the application in the mobile phones. The super admin had to extend the date of password expiry, by logging onto the web portal. This issue was solved by notifying at least a day prior.
One of the significant changes made at the webpage was to ease the workload on the specialists. The initial design had prompted the specialist with "submit" button for each image for their recommendation. This resulted in longer time to clear the cases wherein in few instances there were approximately 20 images for each patient. To address these issues the design was changed to 'submit' report at the end of each patient rather than each image. This significantly reduced the worktime and also ensured the diagnosis were made for all images.
The contrast and orientation of the images could not be changed, and images could not be magnified, which was a minor issue faced by the remote specialists. FHPs captured images at varied distances, and at different angles, making it slightly inconvenient for specialists to diagnose. In addition, presence of multiple lesions on a single subsite of the oral cavity, gave no option to the specialists to select more than one diagnosis from the drop-down list. In such instance the additional diagnoses had to be entered in the free-text field.
The smartphones used for screening had no Subscriber Identification Module (SIM) card, therefore the date was not auto updated on a day-to-day basis. The healthcare worker would unknowingly select the default date on the phone, while obtaining the informed consent of the patient. Thus, there was an issue of incorrect date selection, which was immediately addressed by the technical team. The team sorted the issue by marking the correct date as 'default date' on the calendar, and the healthcare worker was required to confirm whether the right date was selected. This process had to be manually checked while recording the first case for each day. The challenges that we faced during the study was not anticipated in advance, but were eventually solved.

2.Developing the deep learning algorithm

2.1 Image pre-processing

An adaptive histogram equalization method is used to improve the quality for the images with low brightness and contrast [1]. We generated a combinatorial three-channel data set that combines the information of AFI and WLI of intraoral probe images for analysis. For the new three-channel dataset, blue channel of the WLI was excluded since a long pass filter in front of the CMOS sensor that blocks the excitation wavelengths (405nm); The normalized ratio of AFI-red and green channels was used because it contains information related to the loss of fluorescence signal and is correlated with OPML and malignant lesions. The green, red channels from the WLI and the normalized ratio of AFI- red and green channels were fused to train both neural networks mentioned below [1].

2.2 Mobile-based algorithm for real-time classification

For the development of the algorithm, we first built a CNN (MobileNet [2]) model which can be implemented on a smartphone device in real-time. MobileNet operates by converting the standard convolutional layers to a more efficient format, depth-wise separable convolution. Depth-wise separable convolution is small, has low-latency, and low power consumptions, characteristics which meet the needs of real-time high accuracy analysis for on-device embedded applications. The depth-wise separable convolution layer is made of a depth-wise convolution and a point-wise convolution. The depth-wise convolution layer filters each of the input channels and the pointwise convolution layer combines the results through the depth-wise convolution layer. The computational cost and model size are drastically reduced because of the conversion.We used Nvidia 1080Ti GPU to train the model (pre-trained with ImageNet,

learning rate= 0.0001, 32 batch-size, and 300 epochs), which was then converted to TensorFlow Lite format using tflite converter [3] (reduces the file size and introduces optimizations that do not affect accuracy). The finalized lite format model was approximately 16.3MB and ideal for smartphone platform operation. The customized android smartphone application to control the screening device was also implemented with MobileNet based classification approach. The image preprocessing was implemented on the mobile application which uses the OpenCV android library [4]. The user could use the android application to analyze the captured images by the proposed efficient deep learning classification method without an internet connection. The moto G5 android smartphone used for the platform has an octa-core 1.4GHz CPU, Adreno 505 GPU, and 2 GB RAM, which takes approximately 306 milliseconds to process one image pair use CPU and 288 milliseconds with GPU. The model could achieve faster-running speed if using a more advanced smartphone (41ms per image pair on Huawei P30 pro CPU and 18ms on Huawei P30 pro GPU). The performance of MobileNet was compared with InceptionV3 and VGG19 CNN models, which significantly reduced the number of parameters and model size with minimal accuracy compromises.

2.3 Bayesian deep learning model with uncertainty estimation

We developed another CNN based on the Bayesian deep learning (BDL) [5] framework. Bayesian deep learning is an effective method to add uncertainty handling in deep learning models, as it combines Bayesian probability theory with deep learning to extend standard neural networks by assigning distributions to their weights. Standard deep networks have fixed weights while the weights of Bayesian networks are assigned a probability distribution. Therefore, a standard deep network with fixed weights will always give the same outputs, but a Bayesian neural network will give stochastic outputs. Bayesian deep neural networks consider a distribution over network parameters instead of a single best set. The predictive posterior probability distribution calculated using the Bayesian deep learning model for a new instance x^* is:

$$p(y|x^*, X, Y) = \int p(y|x^*, \omega) p(\omega|\mathrm{X}, \mathrm{Y}) d\omega \qquad (1)$$

It is a distribution that describes all possible predictions given the network weights and test instance x^*. The width of the predictive posterior distribution would be able to reflect the model's confidence about a certain prediction.

Dropout can be interpreted as a Bayesian approximation of a Gaussian process, model uncertainty can be obtained from dropout neural network models, which called Monte Carlo (MC) dropout [6]. By running the Monte Carlo dropout network (MCDN) multiple (ρ) times to obtain several stochastic outputs, the final prediction on a test instance (predictive mean) can be calculated using a Monte Carlo integration over ρ samples:

$$p(y|x^*, X, Y) \approx \frac{1}{\rho} \sum_{\rho=1}^{\rho} p\left(y \middle| x^*, \omega^{(\rho)}\right) \qquad (2)$$

The variance of the distributions from the ensembles will be considered as the model uncertainty on the prediction:

$$v = \frac{1}{\rho} \sum_{\rho=1}^{\rho} (p\left(y \middle| x^*, \omega^{(\rho)}\right) - p(y|x^*, X, Y))^2 \qquad (3)$$

We trained the Bayesian deep neural network for dual-modal intraoral images using VGG19 (pre-trained with Imagnet). Two dropout layers with 0.5 rate were applied to the last two fully connected layers to implement the MCDN. The model trained for 300 epochs with an initial learning rate of 0.0001 and decay 5 times by every 20 epochs, batch size 32. We used the

model with the best performance on the validation set as the final one. The model was trained on the high performance computing platform of University of Arizona [7]. The Bayesian deep network model was implemented on the cloud server, which could produce predictions as well as correlated uncertainty value. The predictive means calculated by eq. 2 will be used as final predictions by the Bayesian neural network and the predictive standard deviation calculated by eq. 3 is the associated uncertainty value, ρ was set to 50.

2.4 Focal loss and data augmentation

The intraoral image dataset was used to train the CNN models. This dataset was, however, unbalanced in terms of clinical parameters (site and diagnosis). Therefore, we applied both data and algorithm-level approaches to reduce the influence of the unbalanced dataset. Data augmentation increased the amount of input data by random perturbation including padding, horizontal and vertical flipping, random cropping, and rotating. Usually, data augmentation will act on all the training data to increase training set diversity to make the model adapt to a variety of conditions. Data augmentation is also a commonly used over-sampling method to amplify the minority classes. We augmented the training examples for both models based on the ratios of imbalanced classes to over-sample the dataset.

We also adopted Focal loss [8] to address the imbalance issue when training both two models. By adding a modulating factor $(1 - p_t)^\gamma$ to the conventional cross entropy loss, with tunable focusing parameter γ, and weighting factor α, the focal loss is defined as:

$$\mathrm{FL}(\mathrm{p}_t) = -\alpha_t(1 - \mathrm{p}_t)^\gamma \log(\mathrm{p}_t) \qquad (3)$$

Focal loss weighs the contribution of each instance to the loss based on the classification error. The contribution to the loss decreases if the instance is already classified correctly by the deep learning model. It also weighs the contribution of each class to the loss in a more balanced way.

References

[1] Song, Bofan, et al. "Automatic classification of dual-modalilty, smartphone-based oral dysplasia and malignancy images using deep learning." Biomedical optics express 9.11 (2018): 5318-5329.

[2] Howard, Andrew G., et al. "Mobilenets: Efficient convolutional neural networks for mobile vision applications." arXiv preprint arXiv:1704.04861 (2017).

[3] https://www.tensorflow.org/lite/convert

[4] https://opencv.org/android/

[5] Ghahramani, Zoubin. "Probabilistic machine learning and artificial intelligence." Nature 521.7553 (2015): 452-459.

[6] Gal, Yarin, and Zoubin Ghahramani. "Dropout as a bayesian approximation: Representing model uncertainty in deep learning." international conference on machine learning. PMLR, 2016.

[7] https://it.arizona.edu/service/high-performance-computing

[8] Lin, Tsung-Yi, et al. "Focal loss for dense object detection." Proceedings of the IEEE international conference on computer vision. 2017.
